# Supplementary material for: Regulation of plant immunity through histone H3 β-hydroxybutyrylation-mediated transcriptional control
Source: Nat Commun. 2025 Jul 17;16:6588. doi: 10.1038/s41467-025-61474-x (PMC12271380; doi:10.1038/s41467-025-61474-x)
Supplement: Supplementary file 1 — Supplementary information. [file 41467_2025_61474_MOESM1_ESM.pdf]

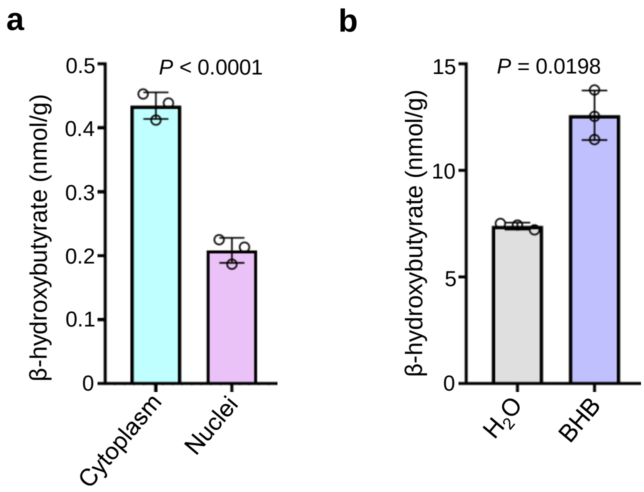

Supplementary Fig. 1. β-hydroxybutyrate (BHB) content analysis in rice. (**a**) BHB content analysis in the cytoplasm and nuclei of rice cells. (**b**) BHB content analysis in rice panicles pretreated with either BHB (100 μM) or H_2O. BHB concentration was assayed by liquid chromatography–tandem mass spectrometry. Data from three independent experiments are shown as mean ± SD. *P* value was calculated by a two-tailed, paired Student *t* test. Source data are provided as a Source Data file.

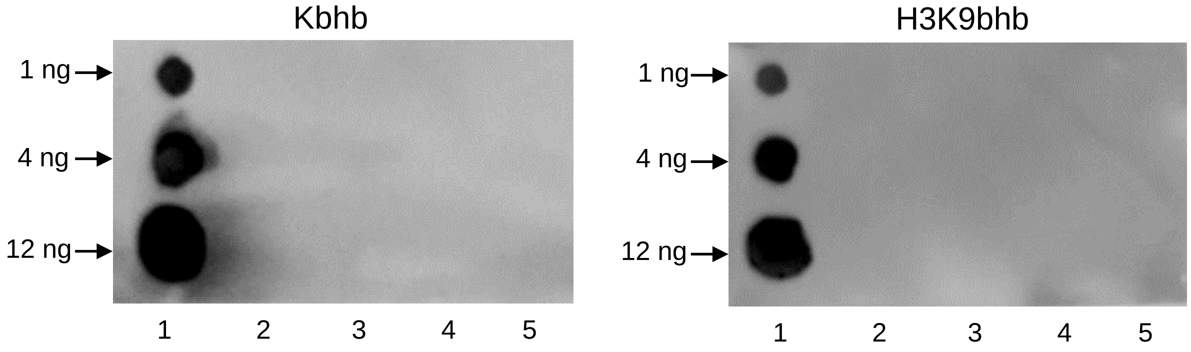

Supplementary Fig. 2. Specificity of Kbhb and H3K9bhb antibodies demonstrated by dot-blot assay. For Kbhb, the peptide samples are as follows: 1: Kbhb; 2: Kac; 3: Kcr; 4: Kla; 5: Khib. For H3K9bhb, the peptide samples are as follows: 1: H3K9bhb; 2: H3K14bhb; 3: H3K9cr; 4: H3K9la; 5: H3K9ac. Images shown are representative of two independent experiments.

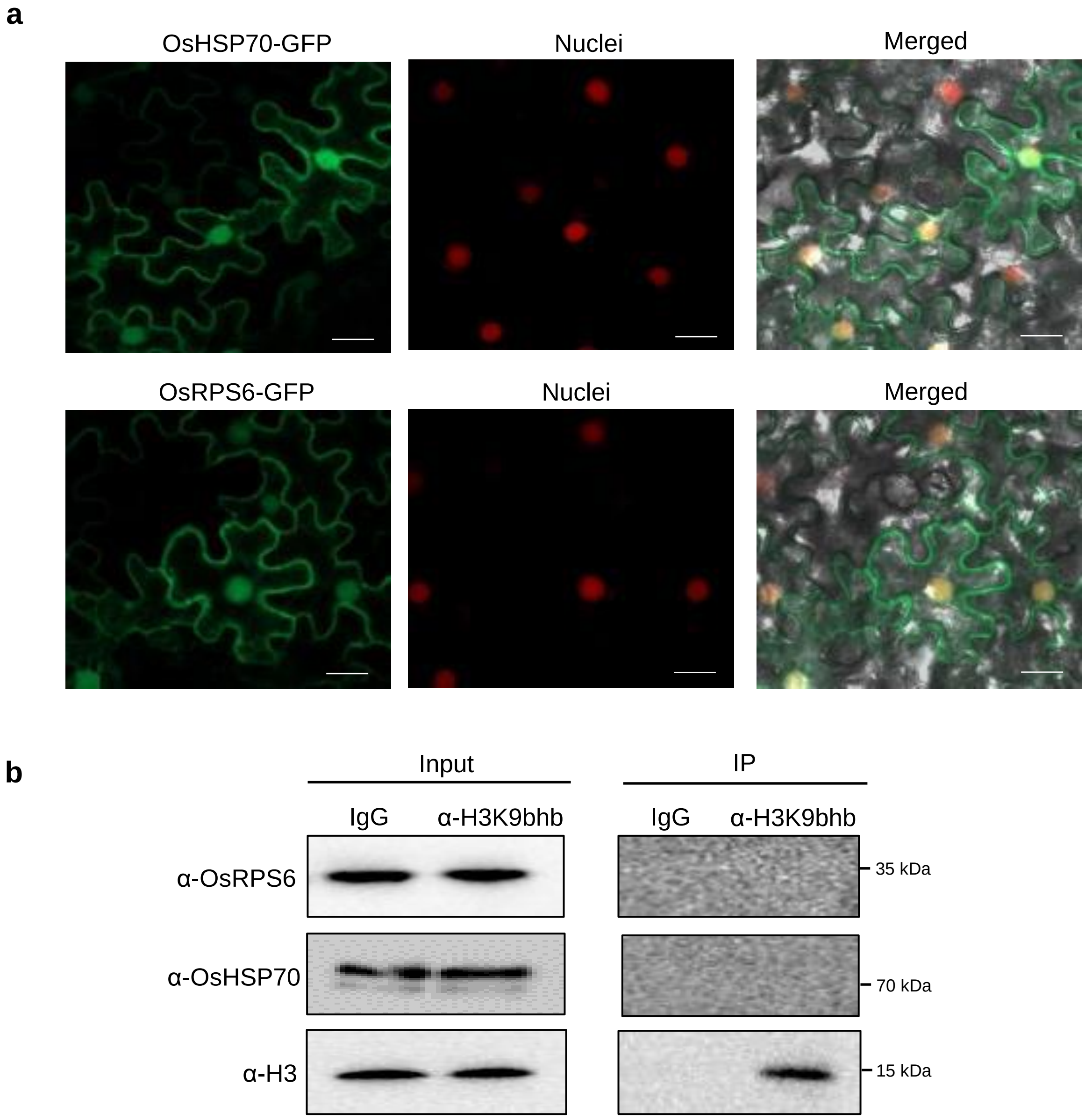

Supplementary Fig. 3. Subcellular localization of OsHSP70 and OsRPS6, and anti-H3K9bhb immunoprecipitation assay. (**a**) Subcellular localization of OsHSP70 and OsRPS6 proteins. Cytoplasmic and nuclear localization of OsHSP70 and OsRPS6 were observed in tobacco leaf cells. OsHSP70 or OsRPS6 coding sequence was fused to GFP at its C-terminus and placed under the control of the cauliflower mosaic virus 35S promoter. RFP signals indicate the nuclei. Scale bars = 20 μm. (**b**) Immunoprecipitation assay using anti-H3K9bhb in rice seedlings. Ncuclear proteins extracted from wild-type rice seedlings were immunoprecipitated with anti-H3K9bhb or IgG (control) and analyzed by immunoblotting with anti-HSP70, anti-OsRPS6, and anti-H3. Anti-H3 was used as a positive control. Images shown are representative of two independent experiments.

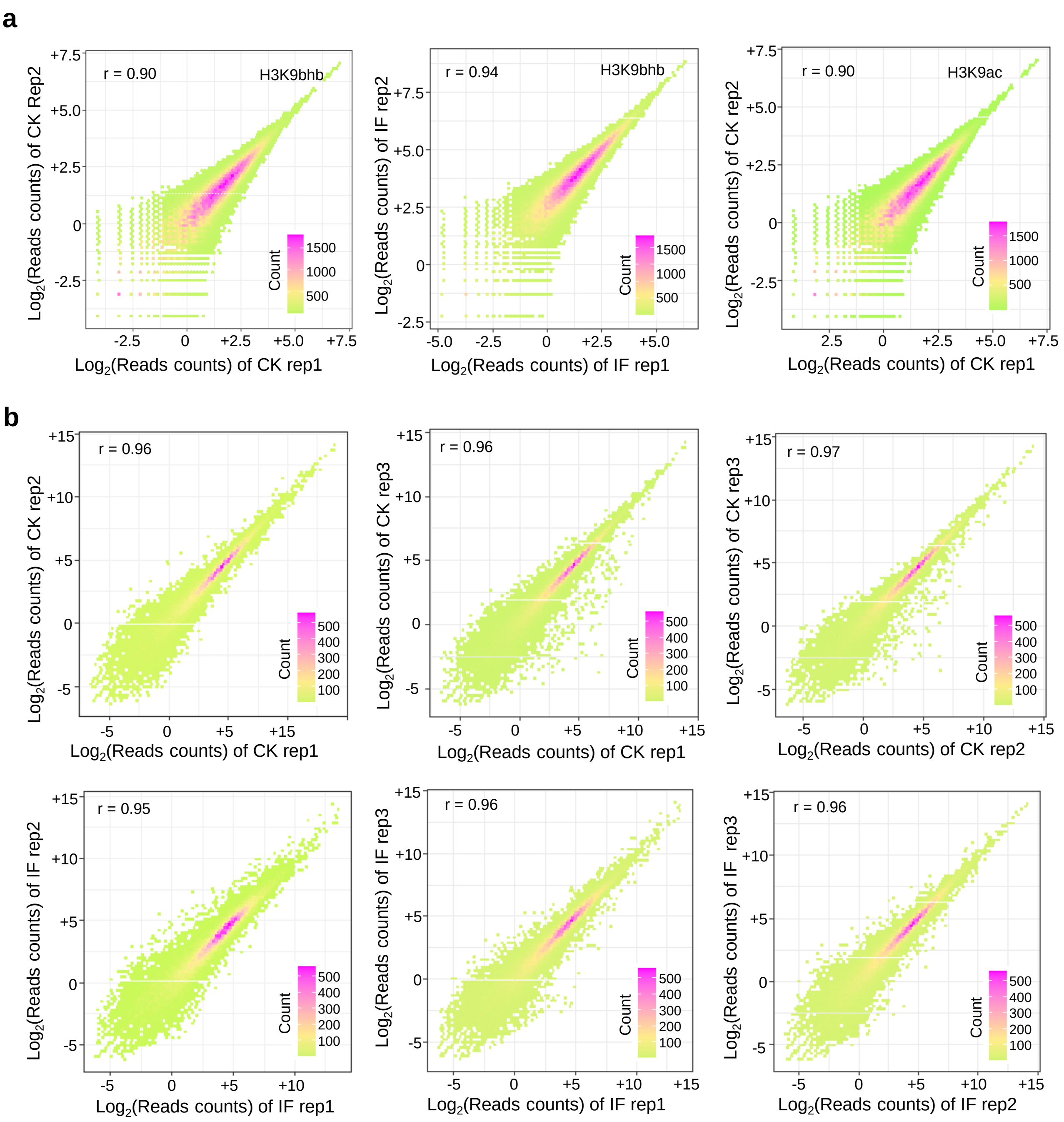

Supplementary Fig. 4. Multiscatter plots of RNA-seq and ChIP-seq data in *U. virens*-infected (IF) and uninfected (CK) rice spikelets at 1 dpi. (**a**) Multiscatter plots of H3K9bhb and H3K9ac ChIP-seq biological replicates (n = 2) for CK or IF rice spikelets. The number of mapped reads from each genomic bin (1 kb) is plotted on a log scale between the two replicates. (**b**) Multiscatter plots of RNA-seq biological replicates (n = 3) for CK and IF rice spikelets. The number of mapped reads for each transcript is plotted on a log scale between the two replicates.

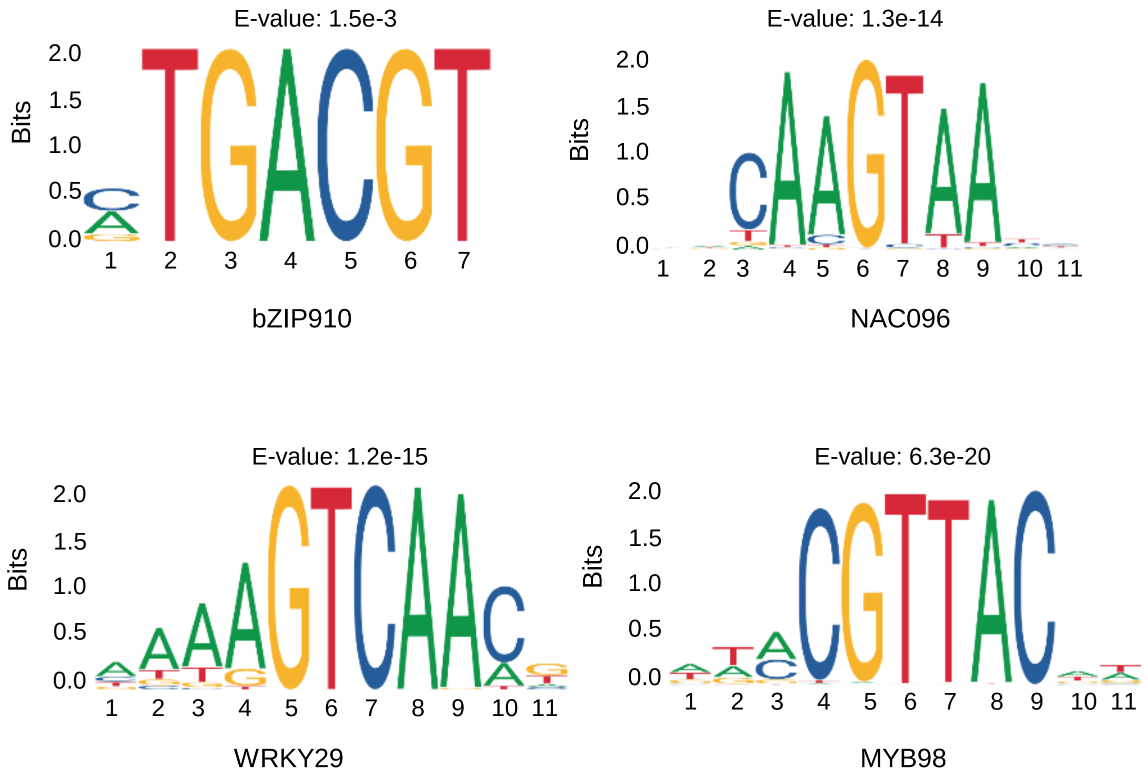

Supplementary Fig. 5. Representative transcription factor binding sites identified in the promoters of H3K9bhb-marked genes.

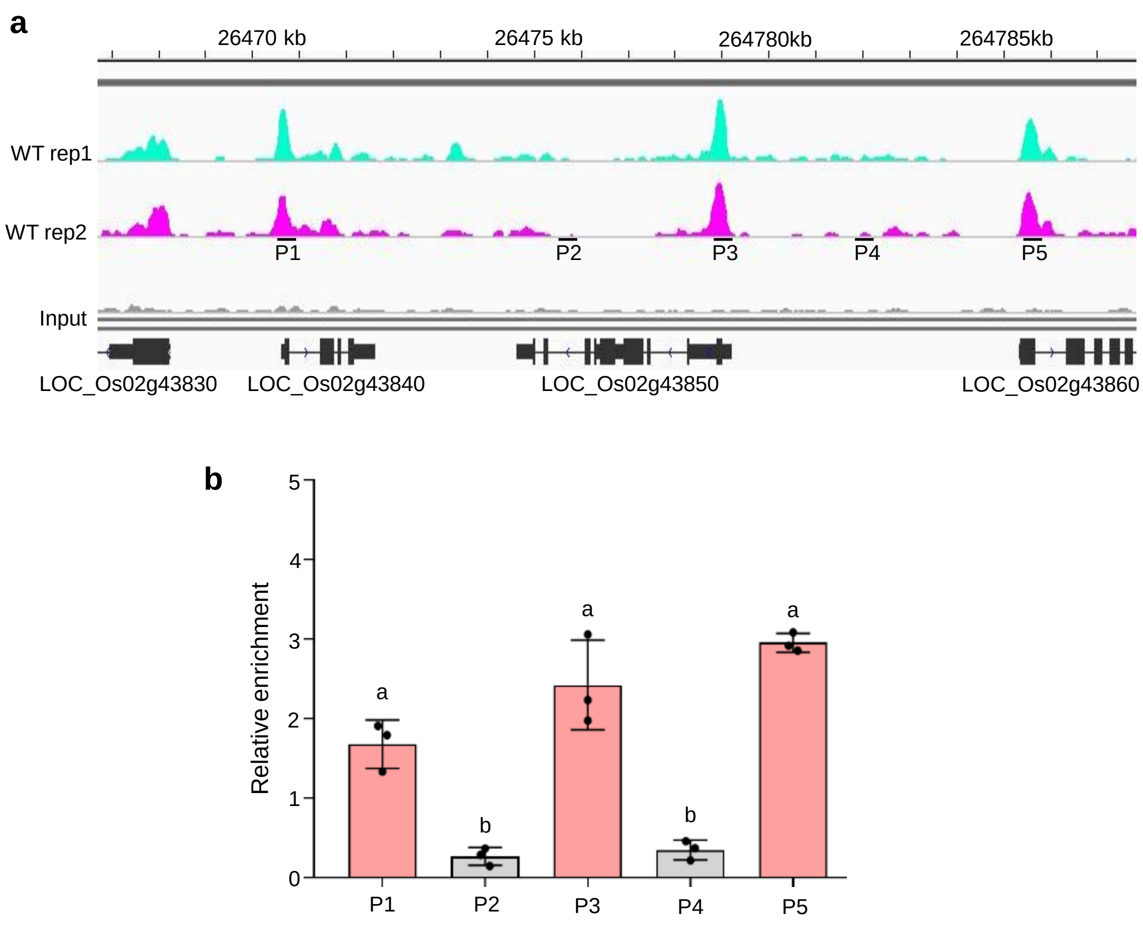

Supplementary Fig. 6. ChIP-qPCR validation of H3K9bhb enriched regions (P1, P3 and P5) or depleted regions (P2 and P4). (**a**) IGV screenshots of selected regions (P1-P5) for tests. IGV, Integrative Genomics Viewer. (**b**) ChIP-qPCR assay of the randomly selected regions (P1–P5). Bars are means ± SD from three biological replicates. Different lowercase letters indicate significant differences at $P < 0.05$. Source data are provided as a Source Data file.

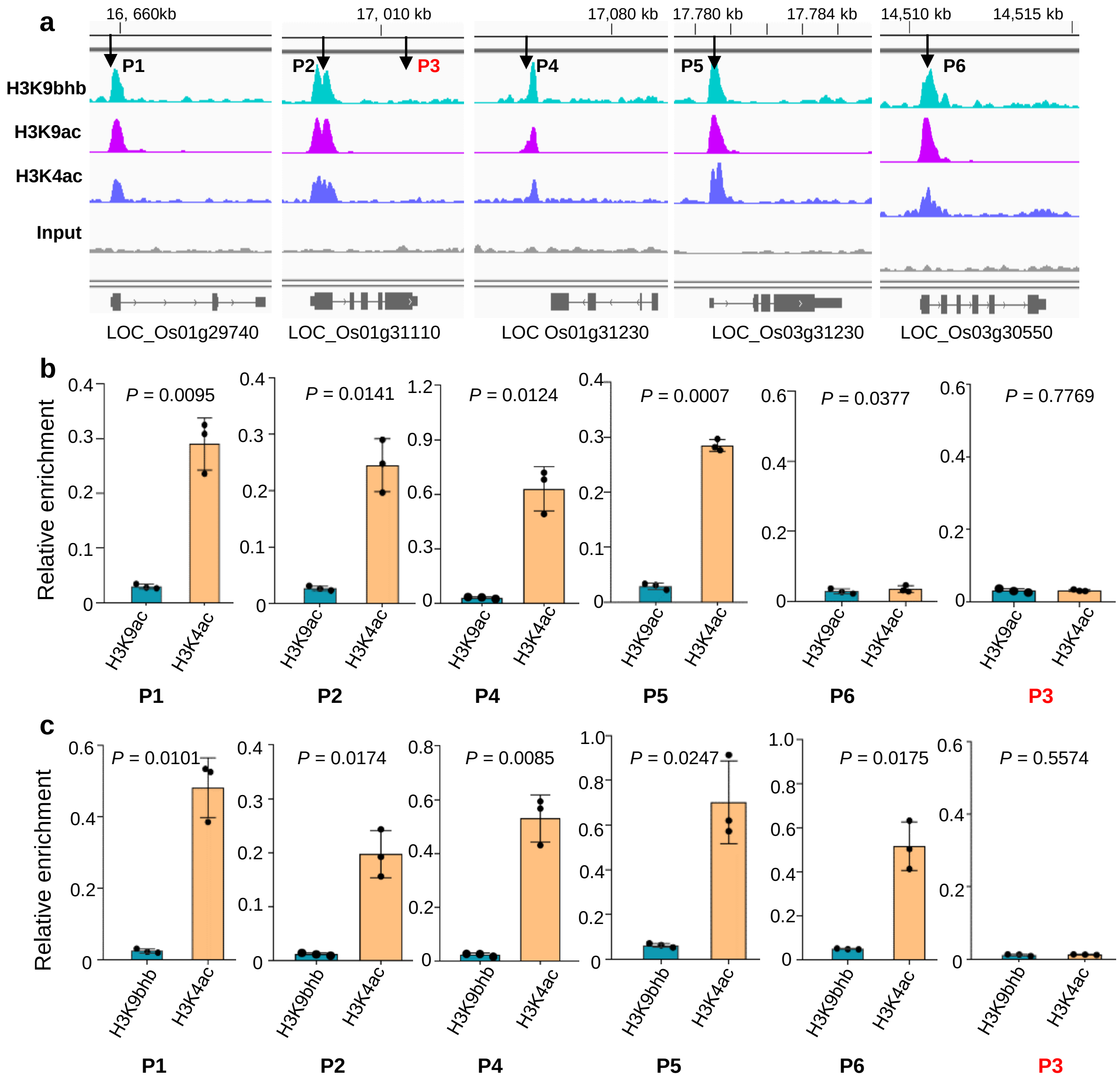

Supplementary Fig. 7. ChIP-reChIP-qPCR analysis of H3K9ac and H3K9bhb. (**a**) Integrative Genomics Viewer (IGV) screnshots showing H3K9bhb and H3K9ac, and H3K4ac peaks. (**b, c**) Sequential chromatin immunoprecipitation (ChIP-reChIP) followed by qPCR analysis was conducted to determine whether H3K9ac and H3K9bhb occupy the same genomic regions. For panel b, chromatin was first immunoprecipitated with H3K9bhb and then re-immunoprecipitated with either H3K9ac or H3K4ac, with H3K4ac serving as a control. For panel c, chromatin was first immunoprecipitated with H3K9ac and then re-immunoprecipitated with either H3K9bhb or H3K4ac, with H3K4ac serving as a control. P3 was used as a non-peak negative control. For all data, bars indicate means ± SD from three replicates. *P* value was calculated by a two-tailed, paired Student *t* test. Source data are provided as a Source Data file.

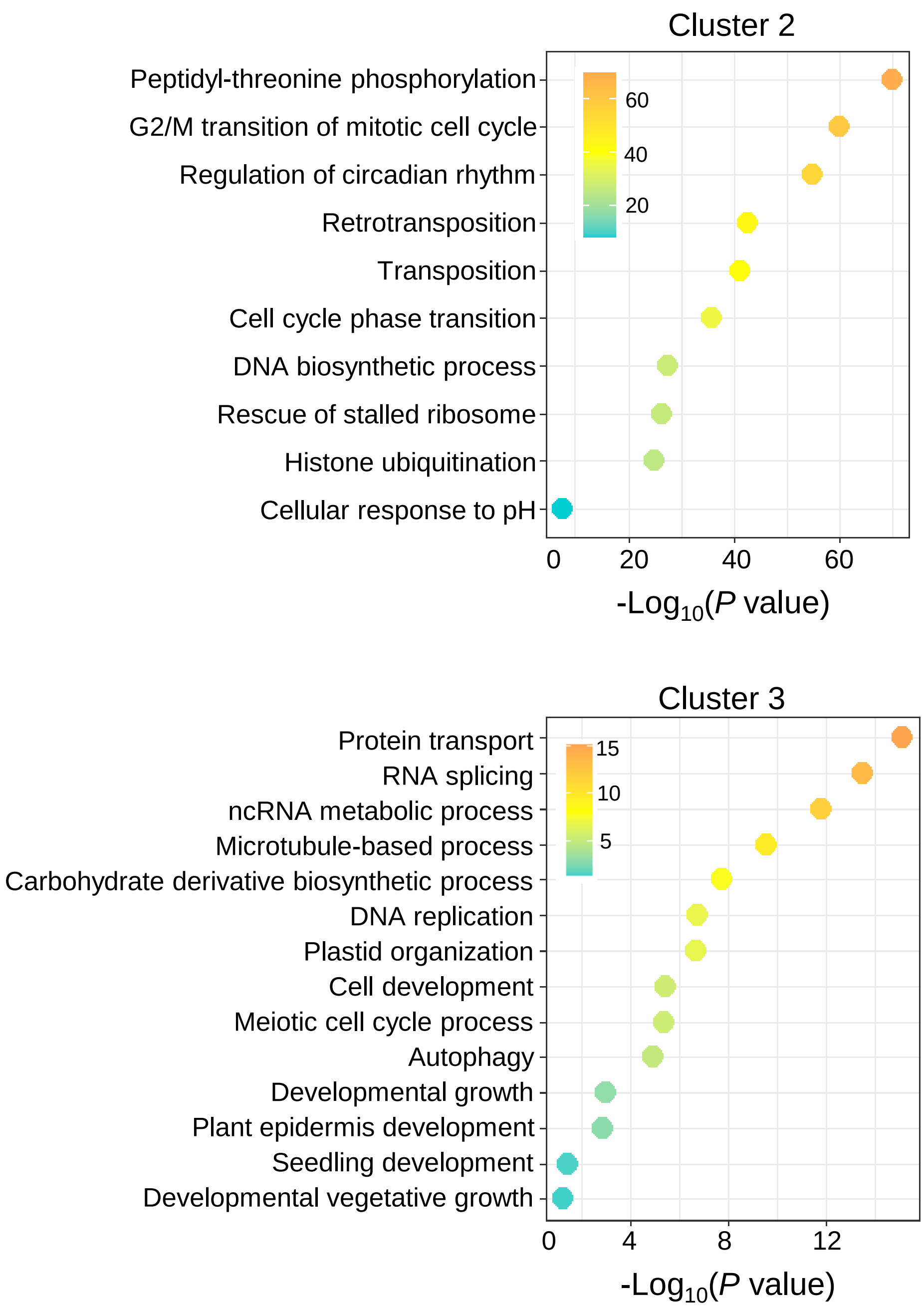

Supplementary Fig. 8. GO pathway enrichment analysis of cluster 2 and 3 genes from Fig. 3a.

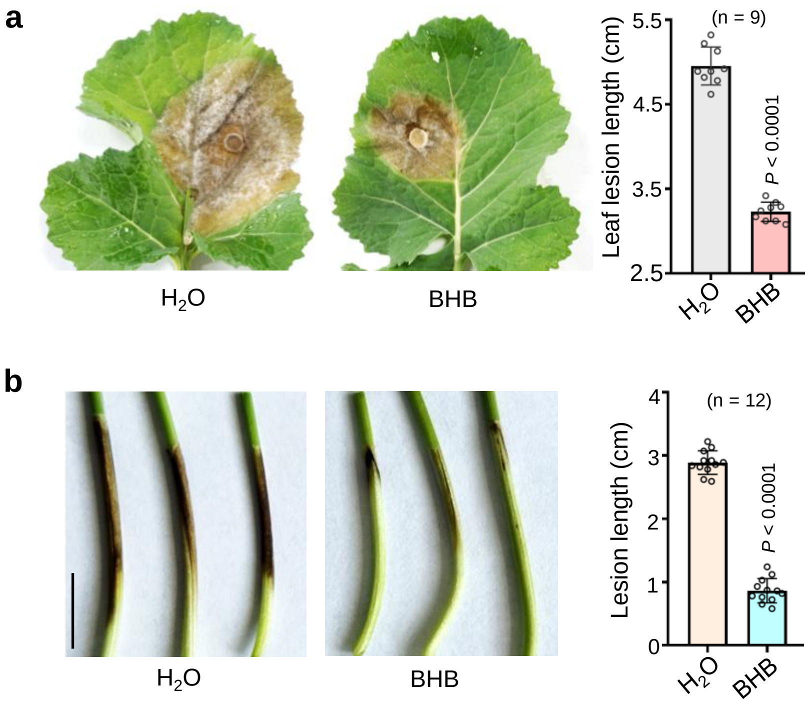

Supplementary Fig. 9. BHB enhances crop resistance to pathogens. (**a**) Rapeseed leaves were treated with H_2O or 100 µM BHB for one day prior to inoculation with *Sclerotinia sclerotiorum* for three days, and lesion lengths were measured. Data were collected from three independent experiments for each treatment with three leaves. (**b**) Wheat coleoptiles were treated with H_2O or 100 µM BHB for one day before inoculation with *Fusarium pseudograminearum* for five days, and lesion lengths were measured. Data were collected from three independent experiments for each treatment with four leaves. Data are presented as means ± SD, *P* value was calculated by a two-tailed, paired Student *t* test. BHB, β-hydroxybutyrate. Source data are provided as a Source Data file.

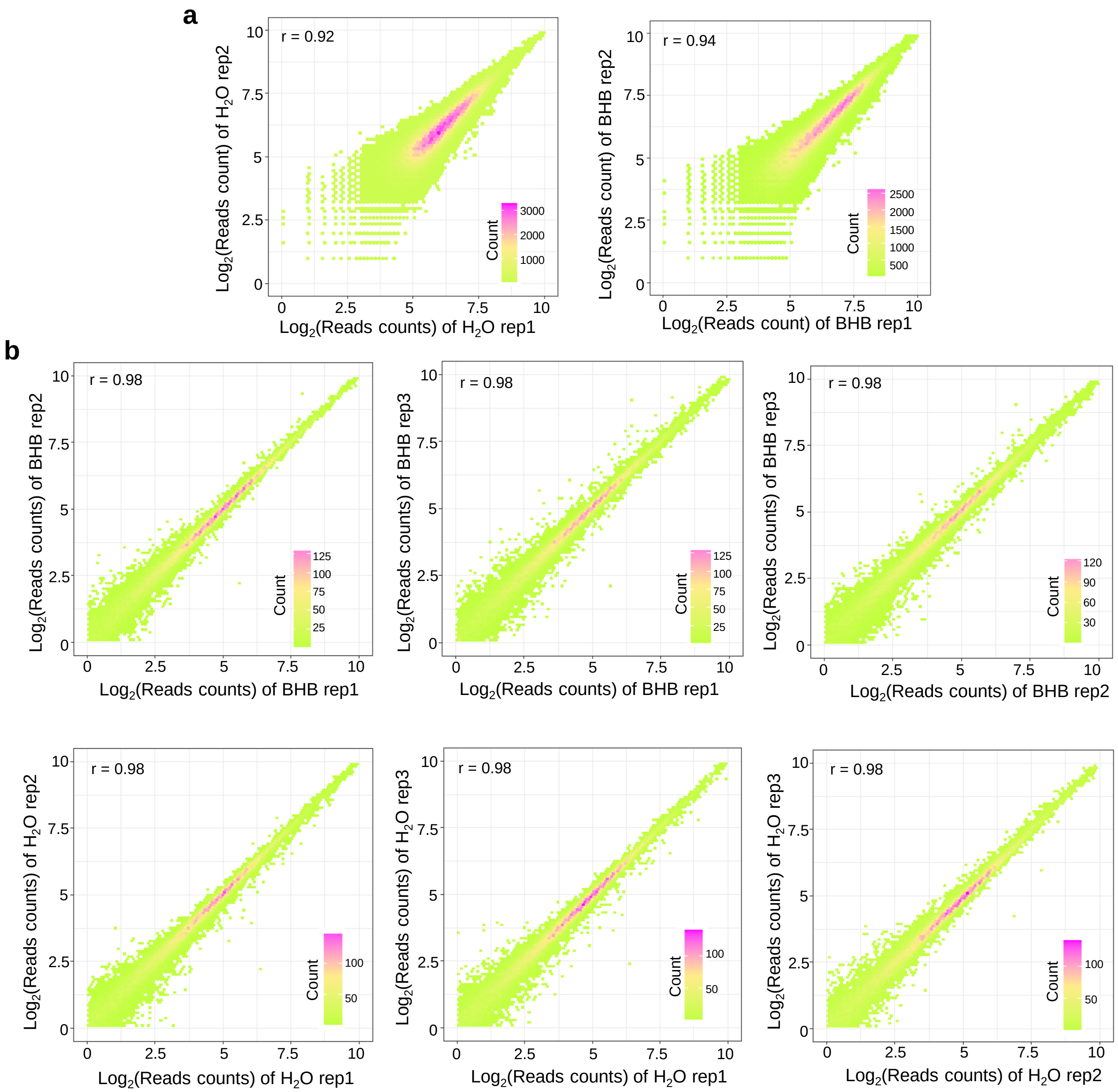

Supplementary Fig. 10. Multiscatter plots of RNA-seq and ChIP-seq data from the water (H_2O) and 100 μM BHB treated rice spikelets. (**a**) Multiscatter plots of H3K9bhb ChIP-seq biological replicates (n = 2) for H_2O and BHB treated rice spikelets. Numbers of the mapped reads from each genomic bin (1 kb) were plotted in log scale between two replicates. (**b**) Multiscatter plots of RNA-seq biological replicates (n = 3) for H_2O and BHB treated rice spikelets. Number of mapped reads for each transcript were plotted in log scale between two replicates. BHB, β-hydroxybutyrate.

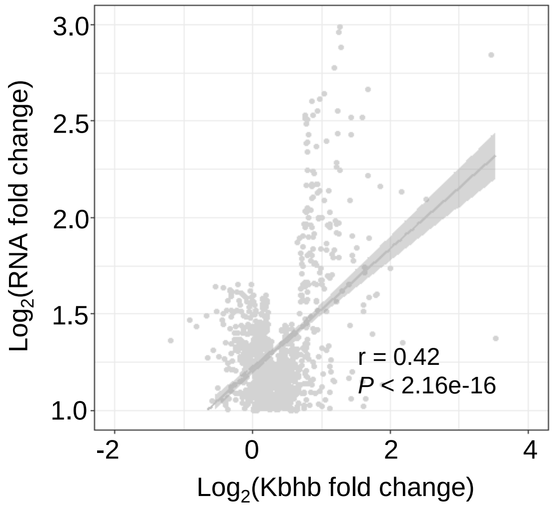

Supplementary Fig. 11. Correlation analysis between expression changes and H3K9bhb modification changes in a subset of genes (n = 1100) in BHB-treated spikelets compared to control (CK) rice spikelets. Pearson correlation coefficient is shown. *P-values* were determined by a two-tailed, paired Student's t-test.

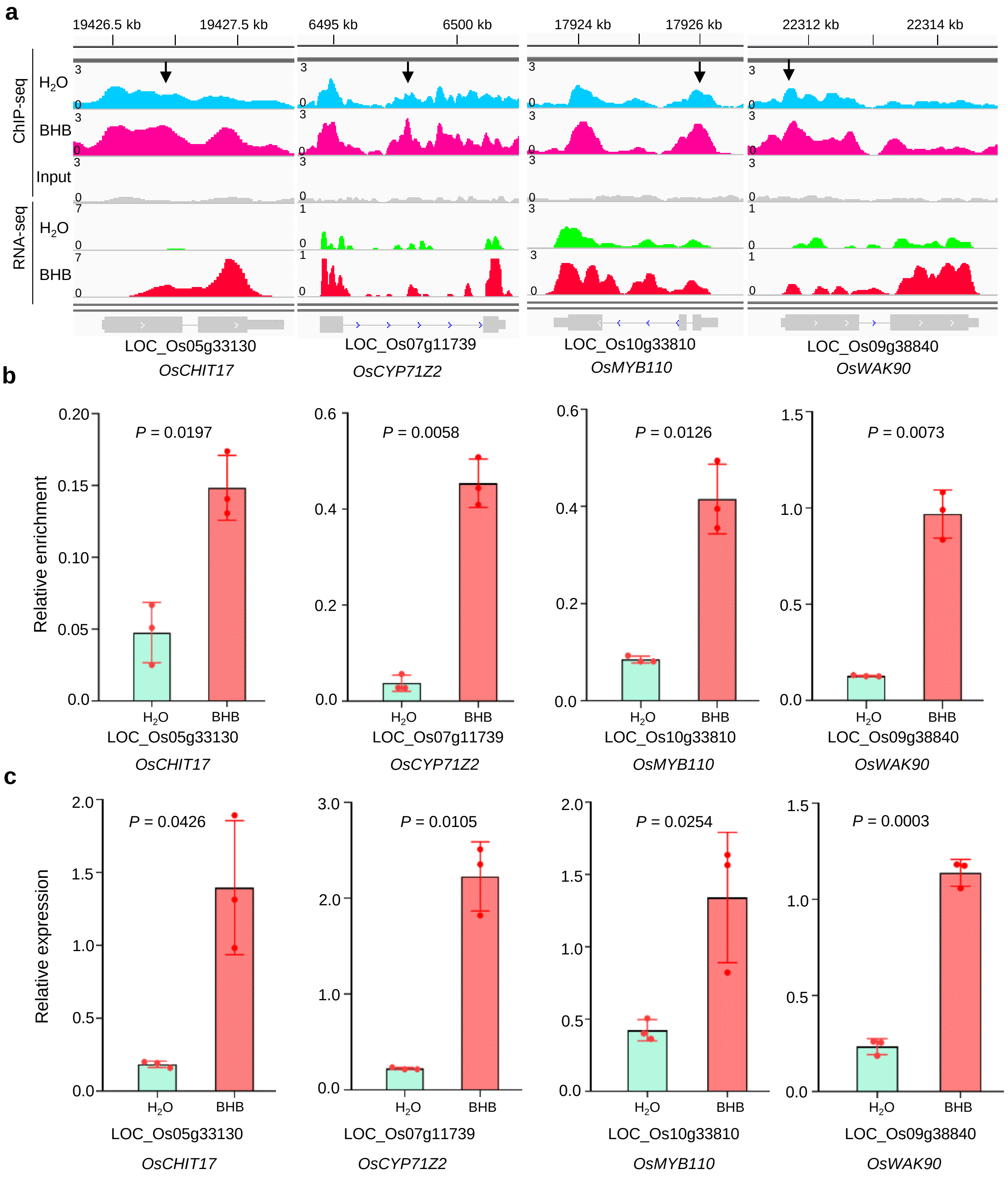

Supplementary Fig. 12. Validation of expression and H3K9bhb levels of genes in the water (H_2O) and 100 μM BHB treated rice spikelets. (**a**) Integrative Genomics Viewer (IGV) screenshots showing H3K9bhb peaks and transcript levels of four genes in H_2O and BHB treated rice spikelets. (**b**) H3K9bhb ChIP-qPCR assays of chromatin isolated from H_2O and BHB treated rice spikelets. (**c**) RT-qPCR analysis of transcript levels in four selected genes in H_2O and BHB treated rice spikelets. For all data, bars indicate means ± SD from three replicates. *P* value was calculated by a two-tailed, paired Student *t* test. BHB, β-hydroxybutyrate. Source data are provided as a Source Data file.

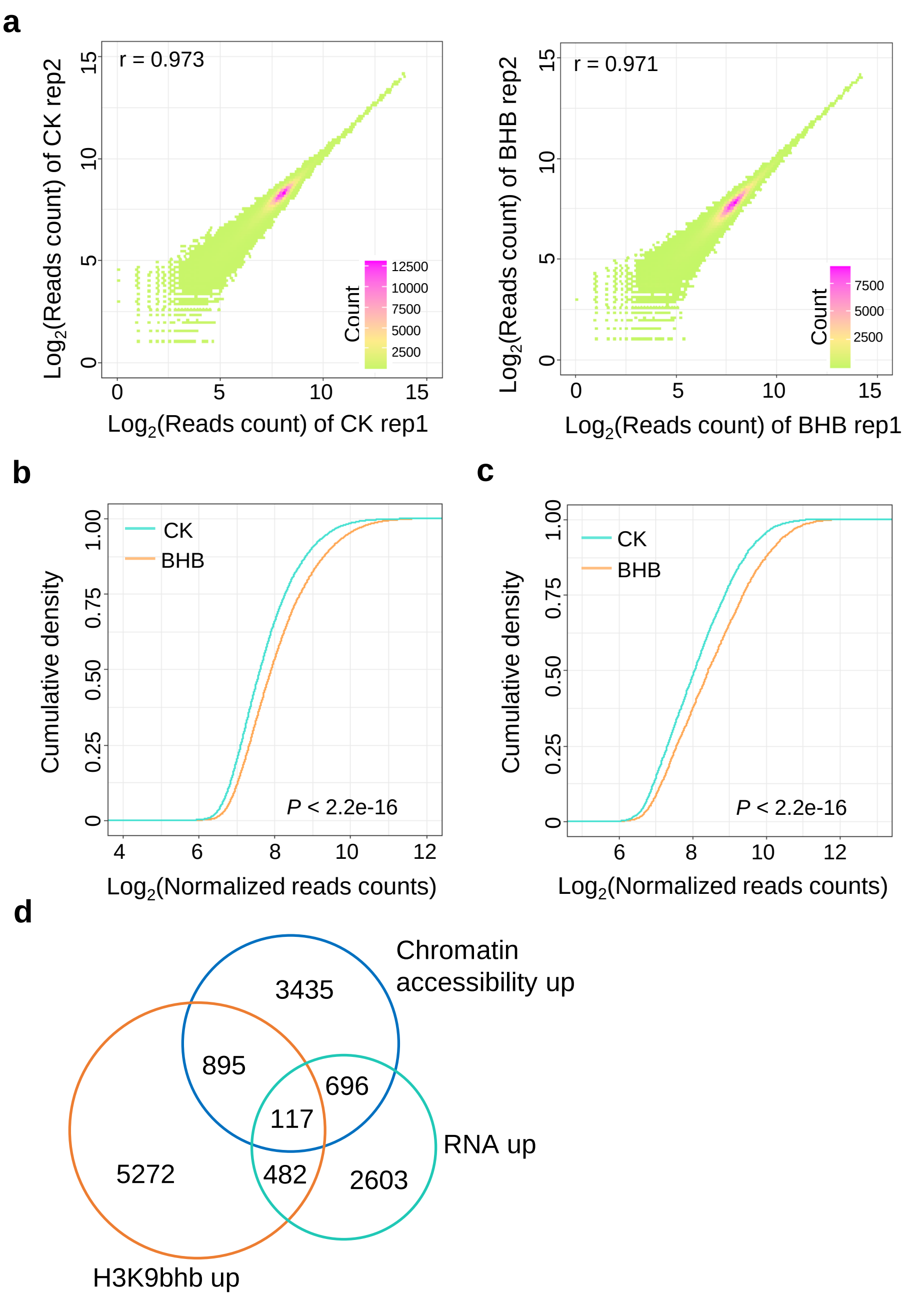

Supplementary Fig. 13. Chromatin accessibility analysis in rice spikelets treated with H_2O (CK) and 100 μM BHB. (**a**) Multiscatter plots of ATAC-seq biological replicates (n = 2) for CK and BHB treated rice spikelets. Numbers of the mapped reads from each genomic bin (1 kb) were plotted in log scale between two replicates. (**b**) Cumulative density plots of chromatin accessibility levels in rice spikelets treated with H_2O and BHB. The *P* value was calculated using a two-sample Kolmogorov–Smirnov test. The read counts were normalized using the DESeq2 size factor normalization method. (**c**) Cumulative density plots showing chromatin accessibility levels for genes with significantly upregulated H3K9bhb in H_2O and BHB treated rice spikelets. The *P* value was calculated using a two-sample Kolmogorov–Smirnov test. The read counts were normalized using the DESeq2 size factor normalization method. (**d**) Venn diagram showing the overlap among Kbhb-upregulated genes, chromatin accessibility-upregulated genes, and transcription-upregulated genes in BHB-treated rice spikelets.

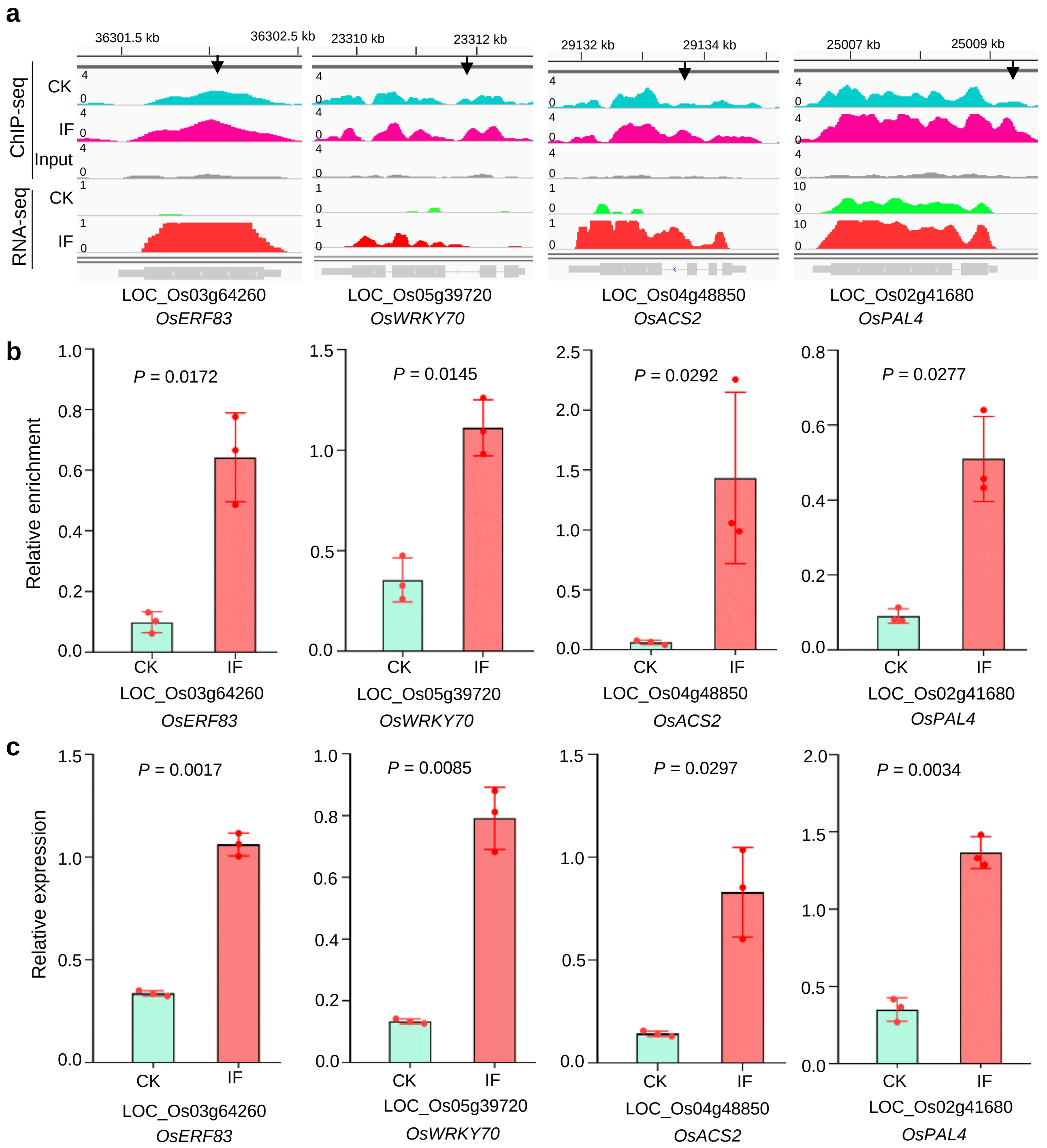

Supplementary Fig. 14. Validation of expression and H3K9bhb levels of genes in *U. virens*-infected (IF) and uninfected (CK) rice spikelets at 1 dpi. (**a**) Integrative Genomics Viewer (IGV) screenshots showing H3K9bhb peaks and transcript levels of four genes in IF and CK rice spikelets. (**b**) H3K9bhb ChIP-qPCR assays of chromatin isolated IF and CK rice spikelets. (**c**) RT-qPCR analysis of transcript levels in four selected genes in IF and CK rice spikelets. For all data, bars indicate means ± SD from three replicates. *P* value was calculated by a two-tailed, paired Student *t* test. Source data are provided as a Source Data file.

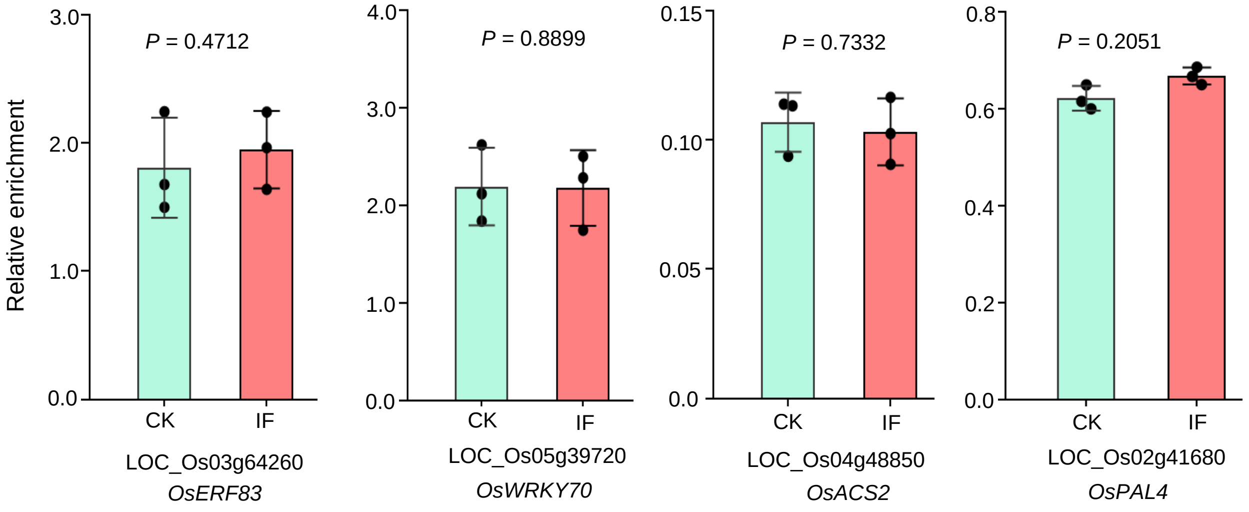

Supplementary Fig. 15. H3K9ac ChIP-qPCR assay of chromatin isolated from *U. virens*-infected (IF) and uninfected (CK) rice spikelets. Bars indicate means ± SD from three replicates. *P* value was calculated by a two-tailed, paired Student *t* test. Source data are provided as a Source Data file.

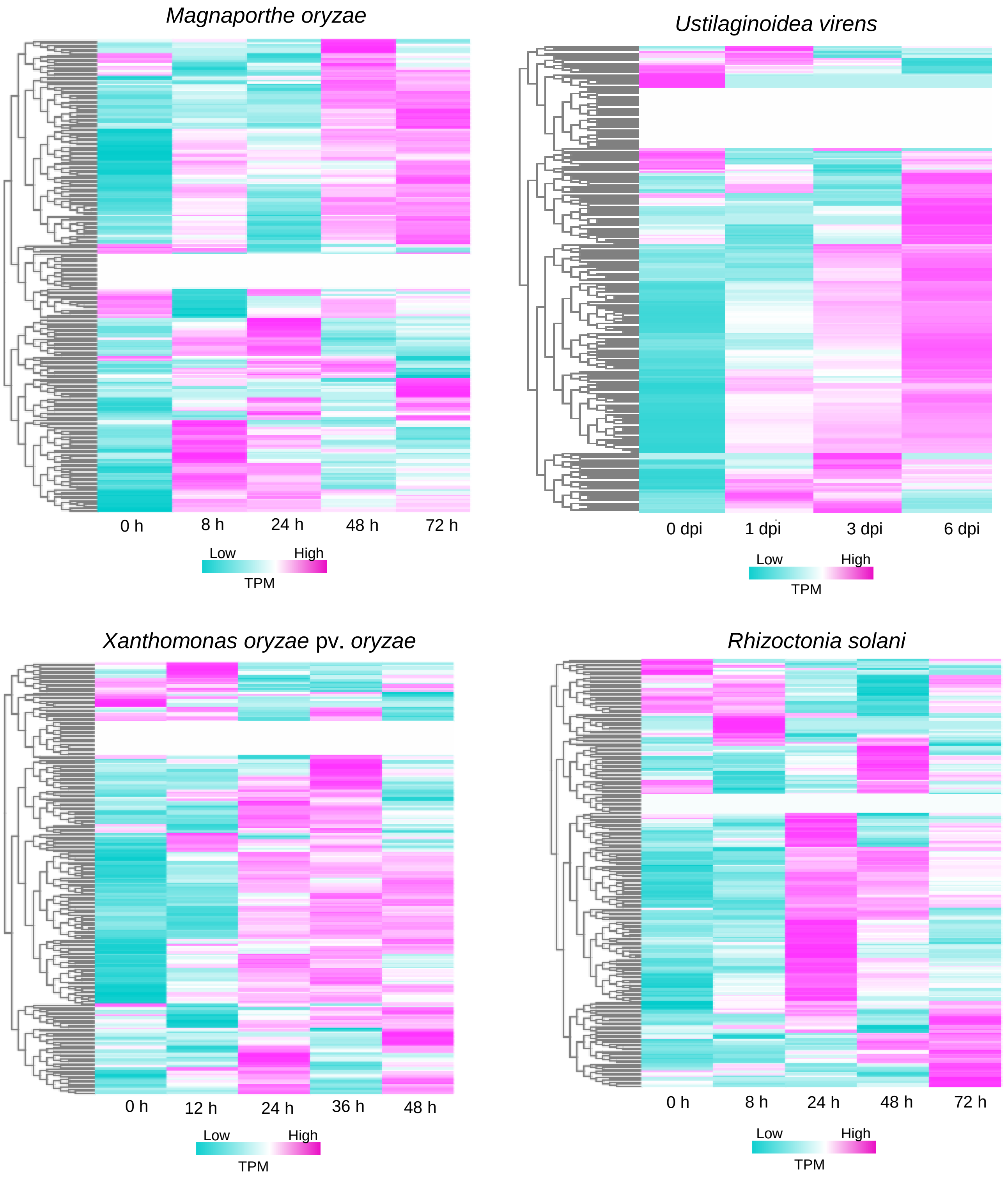

Supplementary Fig. 16. Gene expression analysis of H3K9bhb-hyper and transcriptionally upregulated genes in *U. virens*-infected rice spikelets under different pathogen treatments.

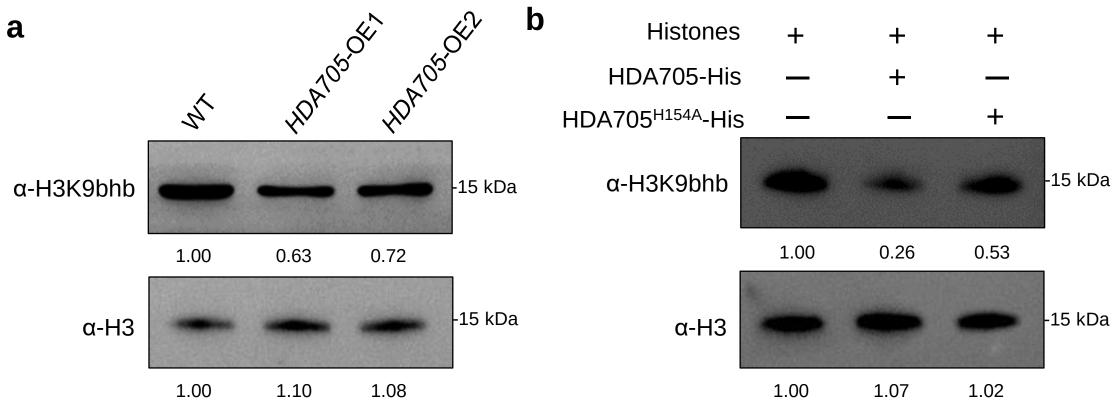

Supplementary Fig. 17. HDA705 activity assay on H3K9bhb. (**a**) Immunoblotting detection of H3K9bhb levels in wild type (WT) plants and *HDA705* overexpression plants. Anti-H3 was used as the loading control. *HDA705* overexpression plants were driven by the cauliflower mosaic virus 35S promoter, with a 3×FLAG tag fused to the N-terminus of the protein. The immunoblot signals were quantified using ImageJ. Relative quantified signals of each band are indicated with the control loading set as 1.00. (**b**) *In vitro* de-H3K9bhb assay of HDA705 and HDA705^{H154A}. Anti-H3 was used as the loading control. A point mutation (H154A) was introduced into the catalytic domain of HDA705. The immunoblot signals were quantified using ImageJ. Relative quantified signals of each band are indicated with the control loading set as 1.00. Images shown are representative of two independent experiments.

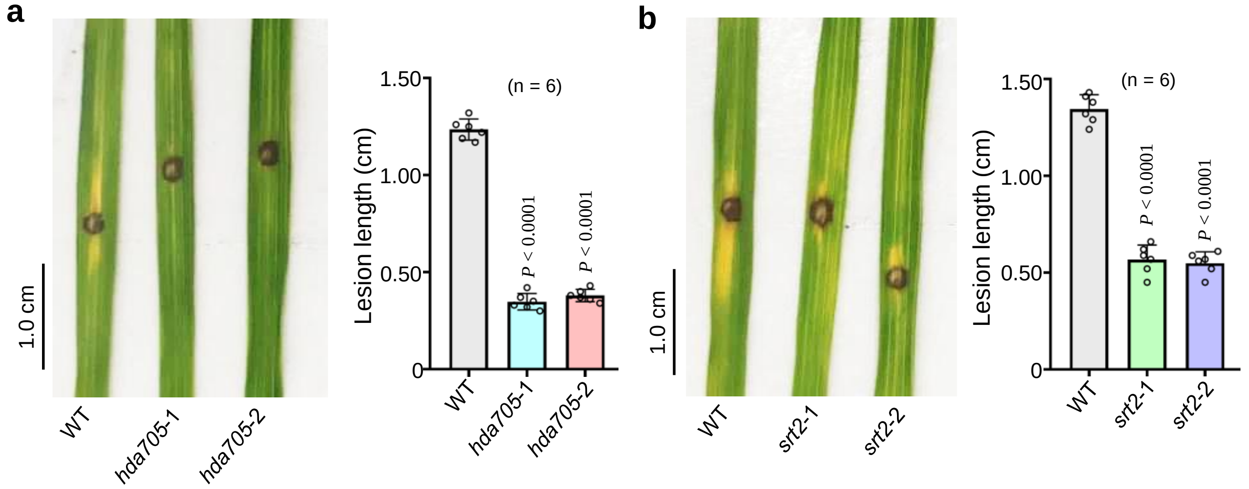

Supplementary Fig. 18. Disease symptoms and lesion length of wild type (WT), *hda705* (a) and *srt2* (b) plants after spot-inoculation with *M. oryzae* at 7 dpi. Bars indicate means ± SD (n = 6). *P* values were determined using one-way ANOVA followed by multiple comparison test. Source data are provided as a Source Data file.

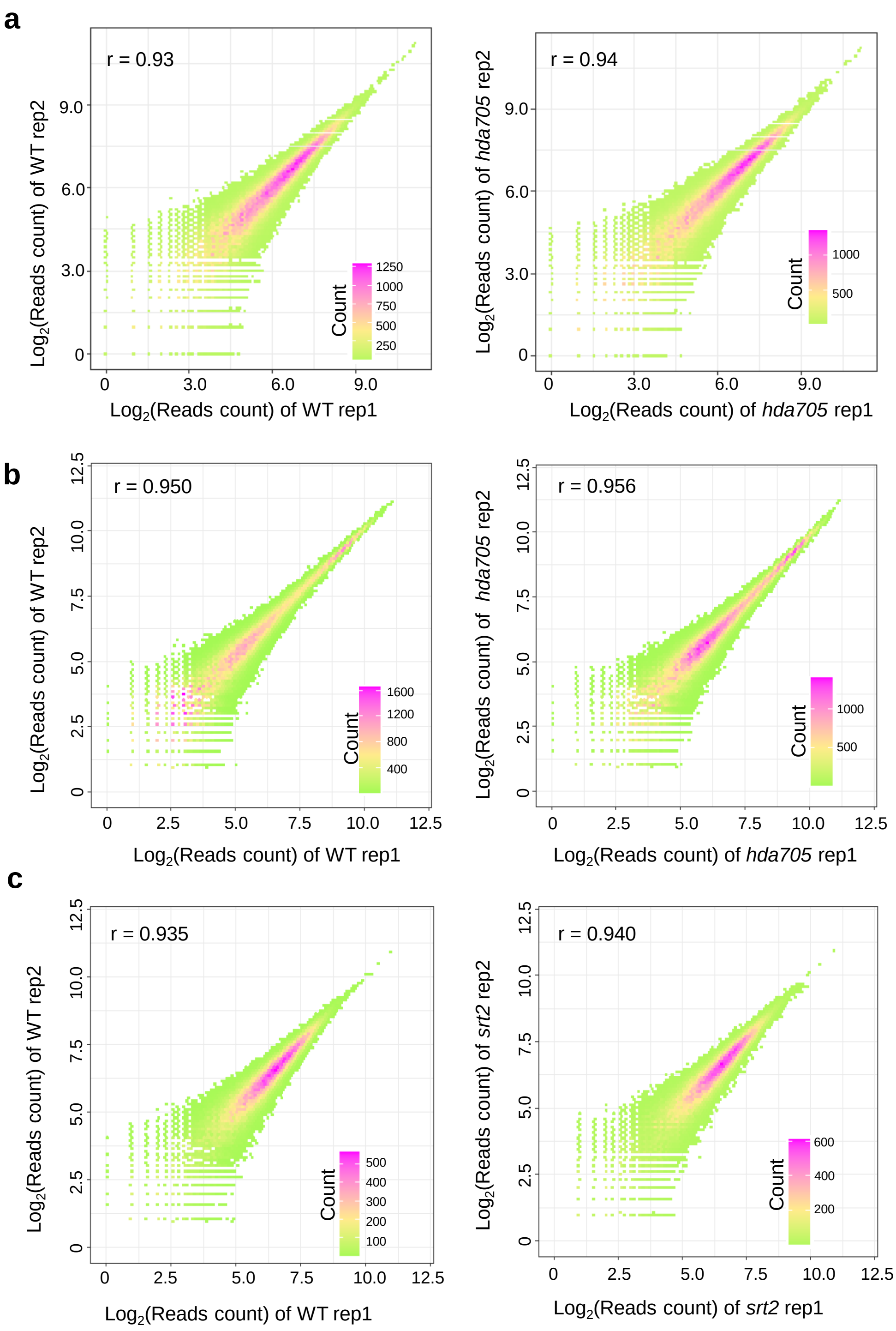

Supplementary Fig. 19. Multiscatter plots of H3K9bhb/H3K9ac ChIP-seq biological replicates for wild type (WT) and *hda705 or srt2* mutant plants. (**a**) Multiscatter plots of H3K9bhb ChIP-seq biological replicates (n = 2) for WT and *hda705* mutant rice plants. Numbers of the mapped reads from each genomic bin (1 kb) were plotted in log scale between two replicates. (**b**) Multiscatter plots of H3K9ac ChIP-seq biological replicates (n = 2) for WT and *hda705* mutant rice plants. Numbers of the mapped reads from each genomic bin (1 kb) were plotted in log scale between two replicates. (**c**) Multiscatter plots of H3K9bhb ChIP-seq biological replicates (n = 2) for WT and *srt2* mutant rice plants. Numbers of the mapped reads from each genomic bin (1 kb) were plotted in log scale between two replicates.

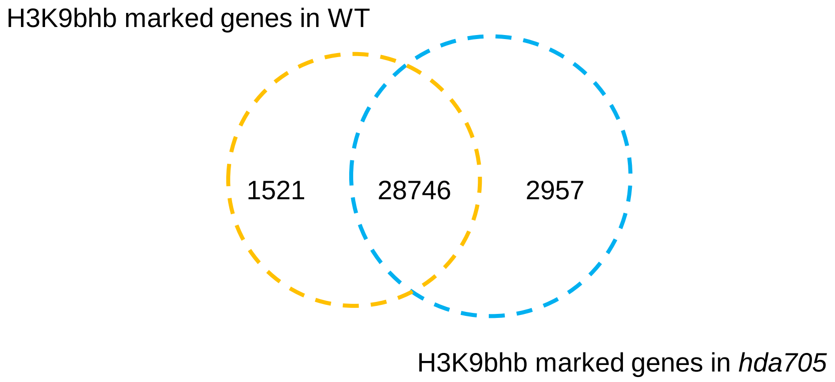

Supplementary Fig. 20. Venn diagram showing the overlap between H3K9bhb-marked genes in *hda705* and wild type plants.

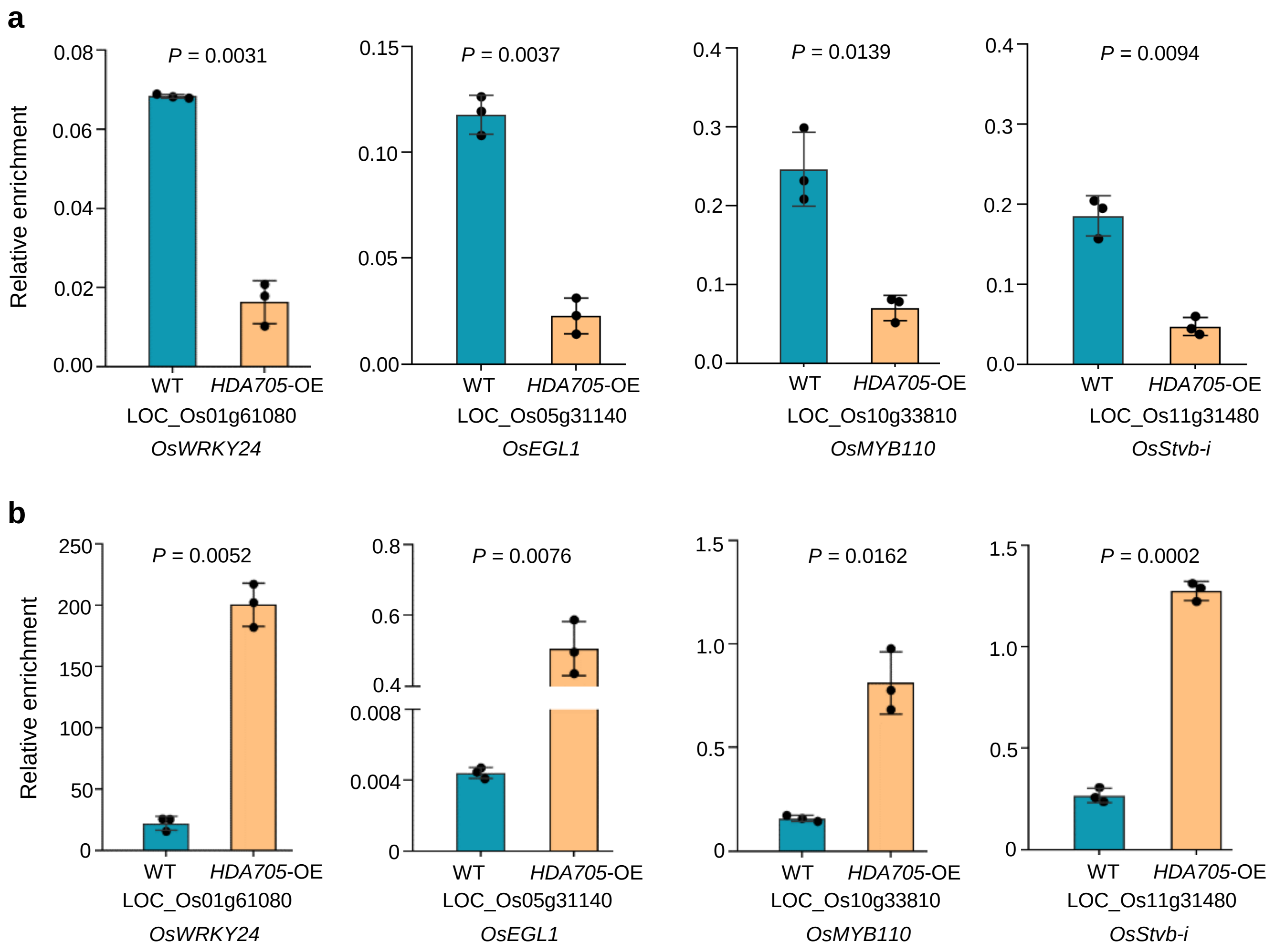

Supplementary Fig. 21. Analysis of H3K9bhb levels and HDA705 binding in wild type (WT) and *HDA705*-overexpression (OE) rice plants. (a) H3K9bhb ChIP-qPCR assay of chromatin isolated from WT and *HDA705*-OE rice plants. **(b)** HDA705 binding ChIP-qPCR analysis of chromatin isolated from WT and *HDA705*-OE rice plants. *HDA705*-OE plants were driven by the cauliflower mosaic virus 35S promoter, with a 3×FLAG tag fused to the N-terminus of the protein. For all data, bars indicate means ± SD from three replicates. *P* value was calculated by a two-tailed, paired Student *t* test. Source data are provided as a Source Data file.

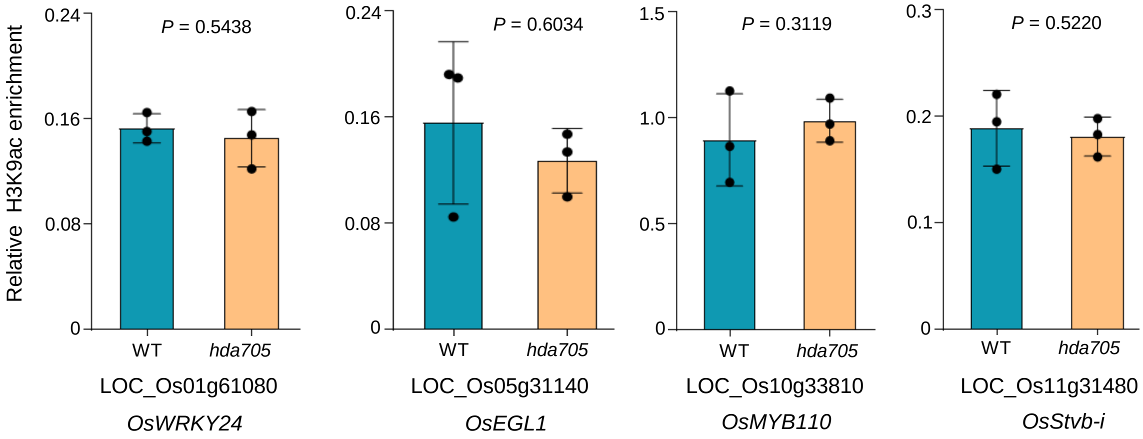

Supplementary Fig. 22. Assay of H3K9ac levels of genes in wild type (WT) and *hda705* plants. For all the data, bars indicate means ± SD from three replicates. *P* value was calculated by a two-tailed, paired Student *t* test. Source data are provided as a Source Data file.

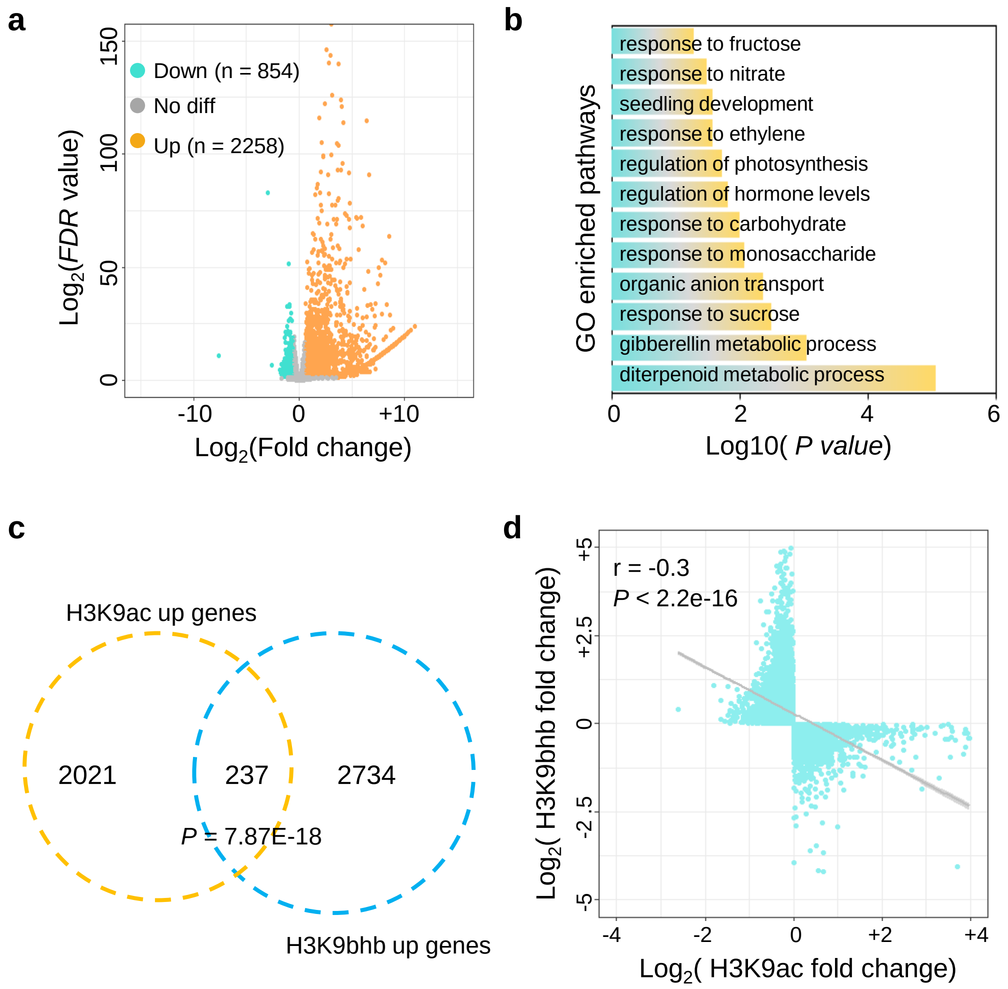

Supplementary Fig. 23. Genome-wide analysis of H3K9ac in wild type (WT) and *hda705* plants. (**a**) Volcano plots of differential H3K9ac levels in *hda705* relative to WT. Orange dots represent upregulated genes (n = 2258, *FDR* < 0.05), light blue dots represent downregulated genes (n = 854, *FDR* < 0.05), and gray dots represent genes with no significant difference. (**b**) GO pathway analysis of the genes (n = 2258) with significantly upregulated H3K9ac levels in *hda705* in comparison to WT. (**c**) Venn diagrams comparing H3K9ac marked genes in WT and *hda705* mutant. *P* value was calculated by Fisher's exact test. (**d**) Correlation analysis of H3K9ac and H3K9bhb changes in *hda705* versus WT rice plants. Genes exhibiting opposite dynamic changes (i.e., increased H3K9ac with decreased H3K9bhb, or vice versa) were selected for the analysis. The Pearson correlation coefficient is shown. The *P* value was calculated using a two-sample Kolmogorov–Smirnov test.

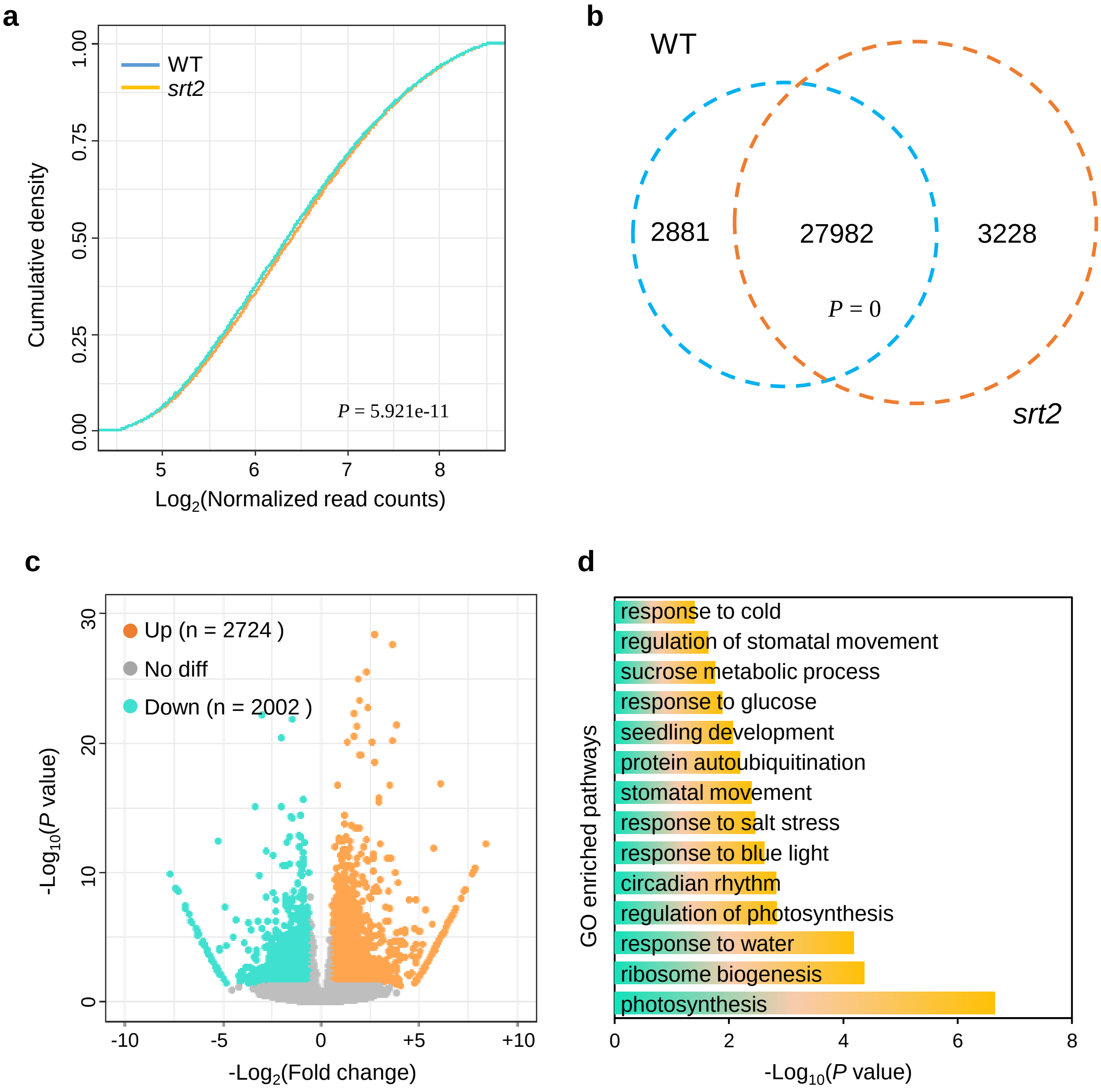

Supplementary Fig. 24. Genome-wide analysis of H3K9bhb in wild type (WT) and *srt2* plants. (**a**) Cumulative density plots of H3K9bhb levels in *srt2* and WT plants. *P* value was calculated from a two-sample Kolmogorov–Smirnov test. The read counts were normalized using the DESeq2 size factor normalization method. (**b**) Venn diagrams comparing H3K9bhb marked genes in WT and *srt2* mutant. *P* value was calculated by Fisher's exact test. (**c**) Volcano plots of differential H3K9bhb levels in *srt2* relative to WT. Orange dots represent upregulated genes (n = 2724, *FDR* < 0.05), light blue dots represent downregulated genes (n = 2002, *FDR* < 0.05), and gray dots represent genes with no significant difference. (**d**) GO pathway analysis of the genes (n = 2724) with significantly upregulated H3K9bhb levels in *srt2* in comparison to WT.

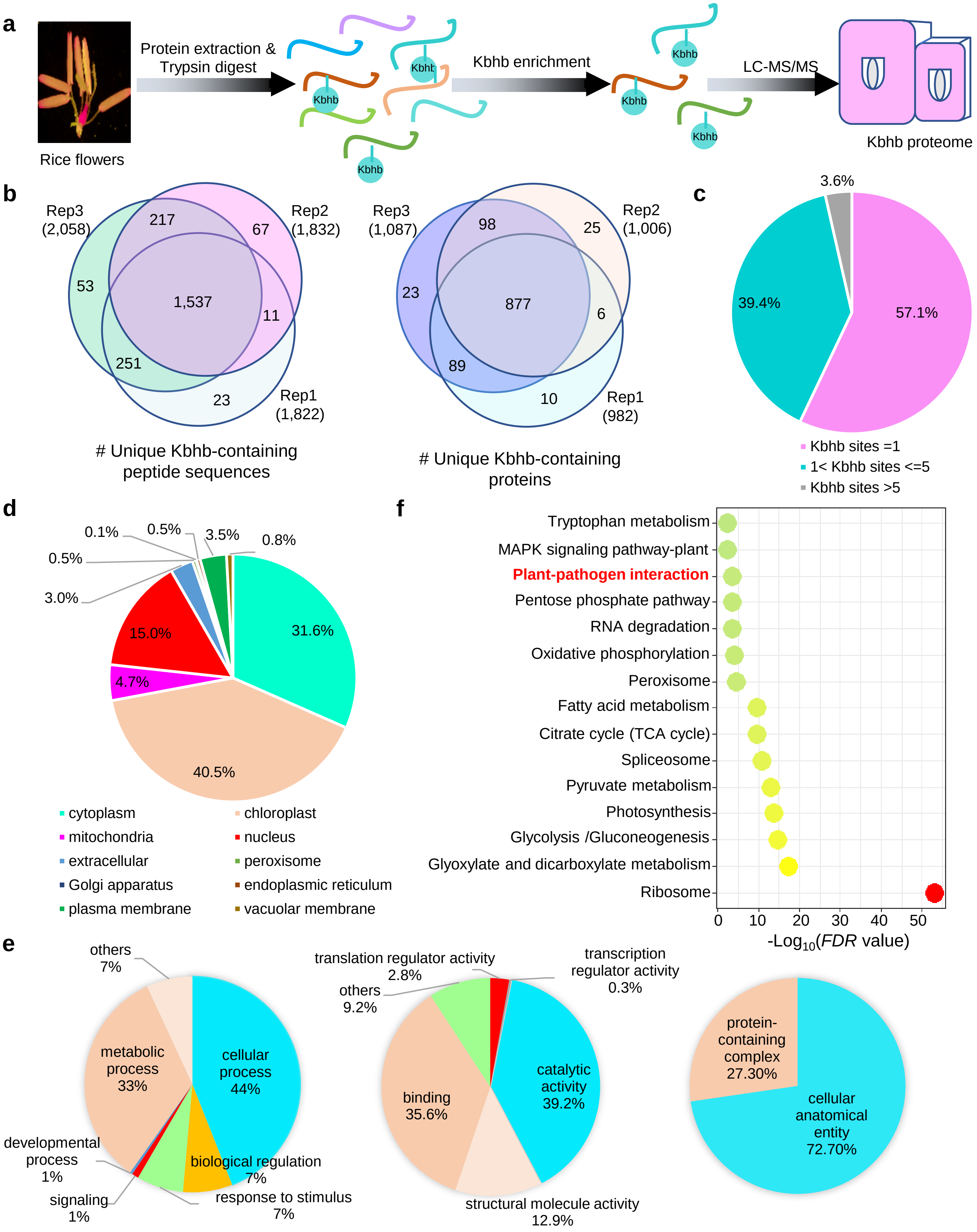

Supplementary Fig. 25. Identification and functional characterization of Kbhb proteome in rice flowers. (**a**) Experimental workflow for the identification of Kbhb proteins. (**b**) Overlap of Kbhb sites and proteins identified across three biological replicates. (**c**) Distribution of the percentage of Kbhb sites per identified protein. (**d**) Pie chart of subcellular localization of Kbhb proteins. (**e**) Gene Ontology (GO) enrichment analysis of Kbhb proteins for the biological processes, molecular functions, and cellular components. (**f**) Kyoto Encyclopedia of Genes and Genomes (KEGG) biological pathway enrichment analysis of Kbhb proteins.

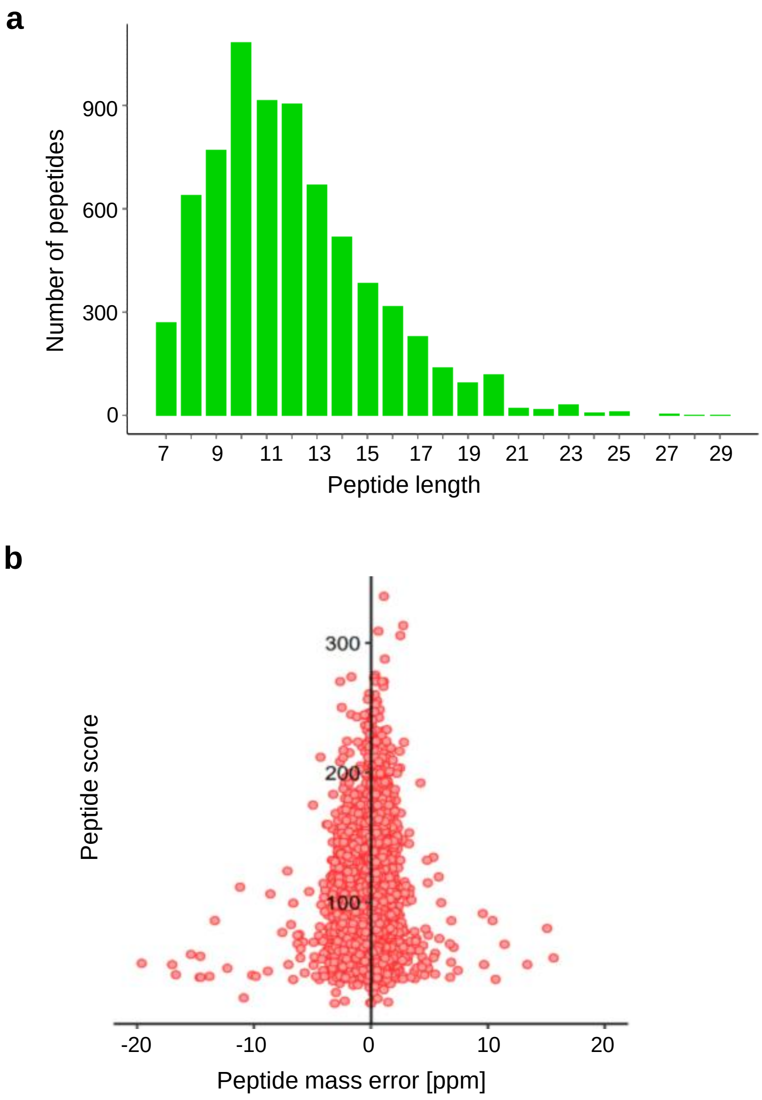

Supplementary Fig. 26. Quality control of MS data. (**a**) Distribution of peptide lengths detected with Kbhb sites by mass spectrometry. (**b**) Mass error of identified Kbhb peptides.

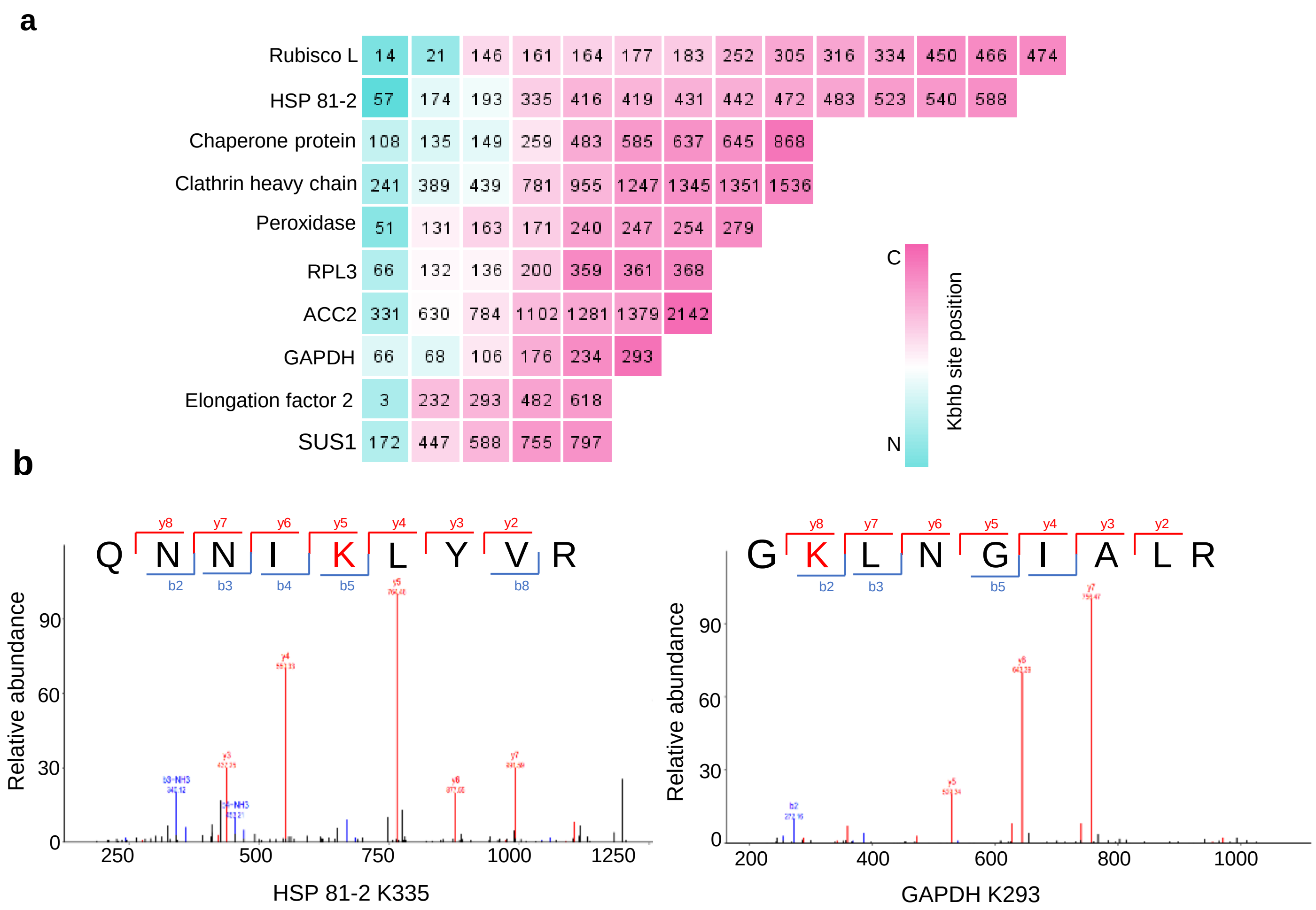

Supplementary Fig. 27. Kbhb sites analysis. (**a**) List of proteins with the most Kbhb sites detected in rice flowers. The number in each square shows position of lysine where the protein was Kbhb modified. Kbhb positions close to the N- and C-terminus of the proteins are shown in cyan and pink, respectively. (**b**) Left: MS/MS spectra of a tryptic peptide for HSP 81-2 Kbhb peptide QNNIK(bhb)LYVR. Right: MS/MS spectra of a tryptic peptide for the GAPDH Kbhb peptide GK(bhb)LNGIALR.

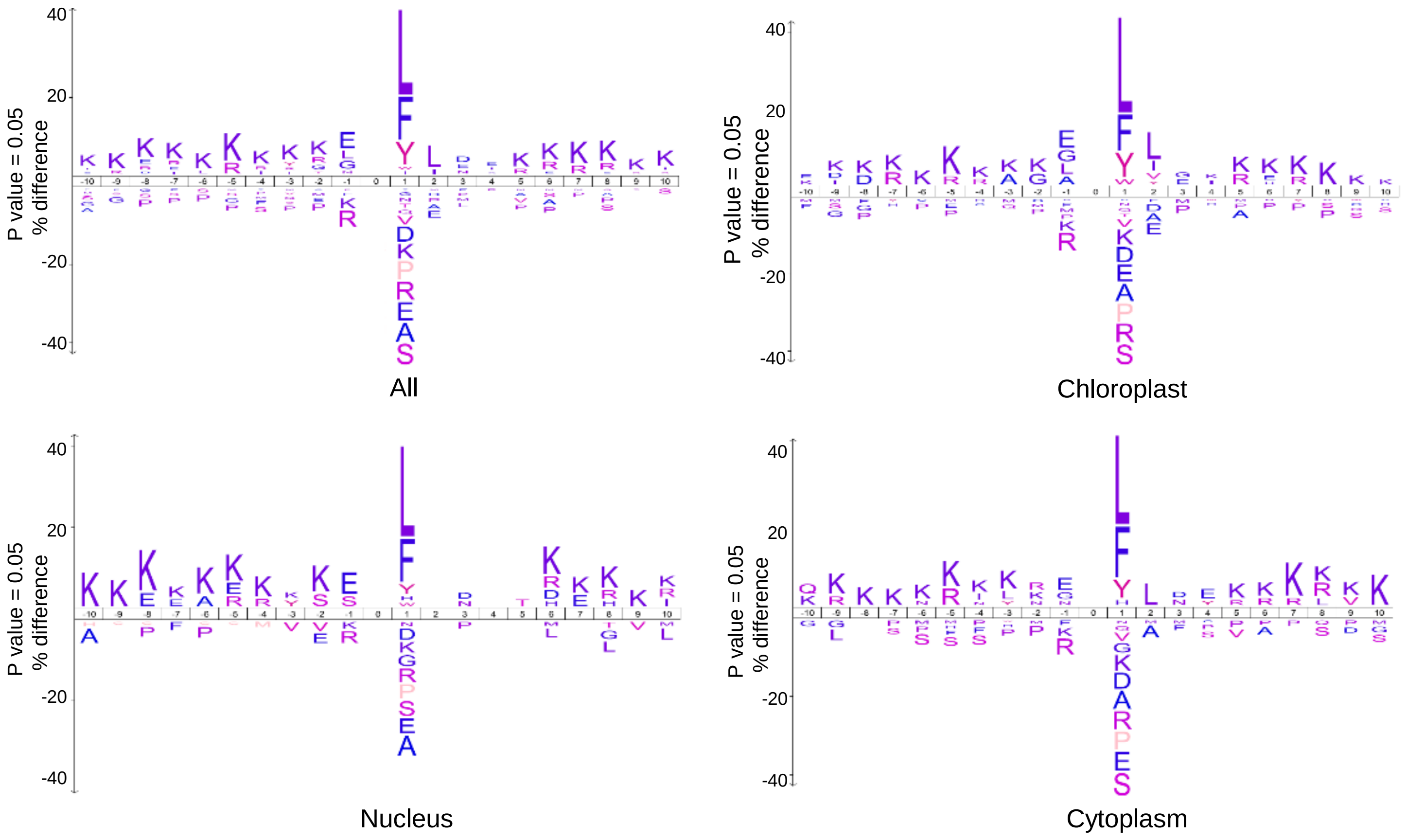

Supplementary Fig. 28. Motif analysis for Kbhb sites in the plastids, cytoplasm, nucleus, and mitochondrion with respective proteins identified in sub-organelles as background. The numbers -10 and +10 indicate the upstream and downstream flanking sequences of lysine (number 0), respectively.

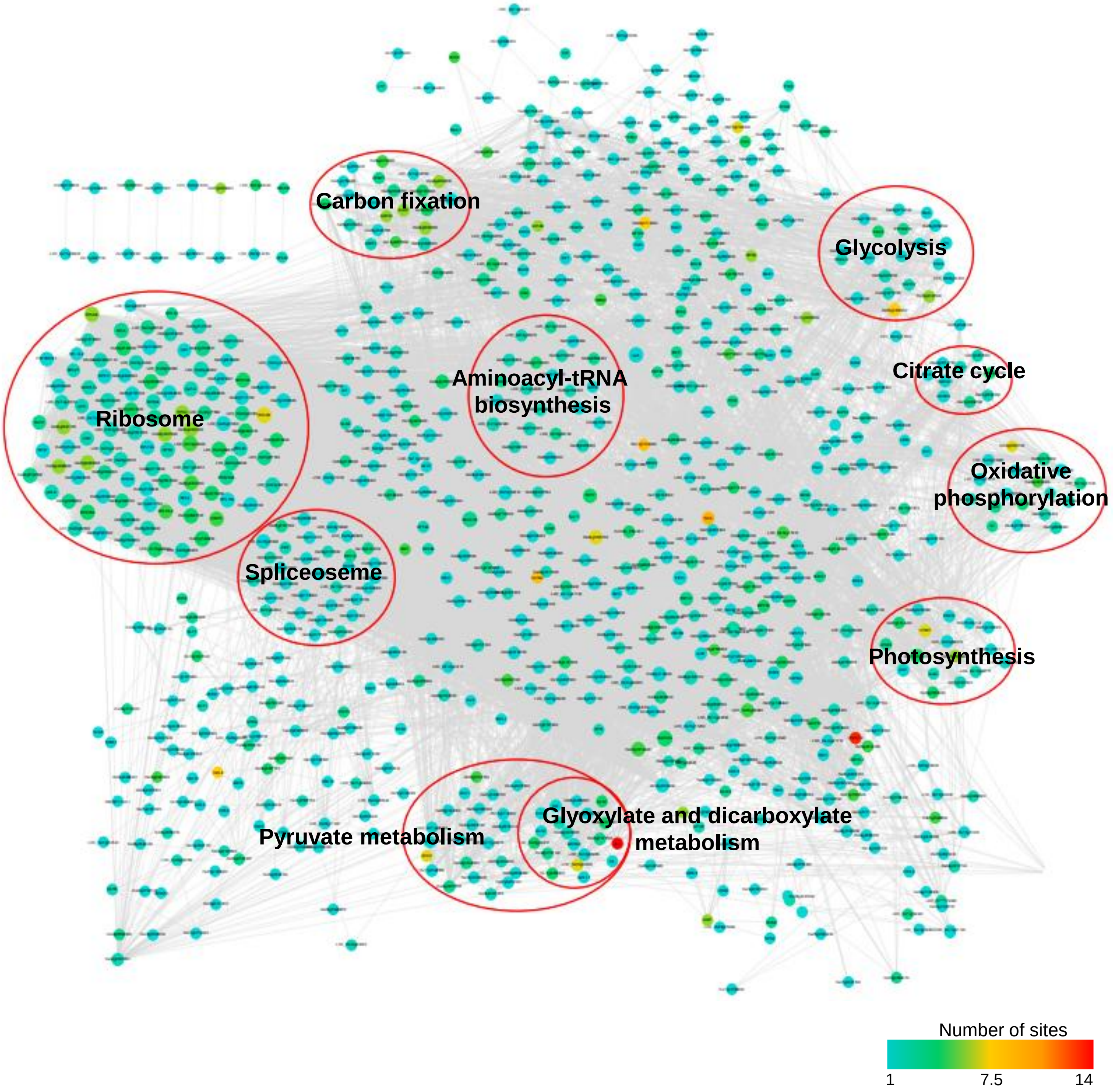

Supplemental Fig. 29. Protein–protein interaction (PPI) map of Kbhb modified proteins identified in rice flowers. Red circles indicate proteins in clusters.
