# Supplementary material for: Regulation of plant immunity through histone H3 β-hydroxybutyrylation-mediated transcriptional control
Source: Nat Commun. 2025 Jul 17;16:6588. doi: 10.1038/s41467-025-61474-x (PMC12271380; doi:10.1038/s41467-025-61474-x)
Supplement: Supplementary file 3 — Reporting Summary [file 41467_2025_61474_MOESM3_ESM.pdf]

nature portfolio

Corresponding author(s): Xiaoyang Chen

Last updated by author(s): Jun 10, 2025

Reporting Summary

Nature Portfolio wishes to improve the reproducibility of the work that we publish. This form provides structure for consistency and transparency in reporting. For further information on Nature Portfolio policies, see our Editorial Policies and the Editorial Policy Checklist.

Statistics

For all statistical analyses, confirm that the following items are present in the figure legend, table legend, main text, or Methods section.

n/a	Confirmed	
☐	☒	The exact sample size (*n*) for each experimental group/condition, given as a discrete number and unit of measurement
☐	☒	A statement on whether measurements were taken from distinct samples or whether the same sample was measured repeatedly
☐	☒	The statistical test(s) used AND whether they are one- or two-sided<br>*Only common tests should be described solely by name; describe more complex techniques in the Methods section.*
☒	☐	A description of all covariates tested
☒	☐	A description of any assumptions or corrections, such as tests of normality and adjustment for multiple comparisons
☐	☒	A full description of the statistical parameters including central tendency (e.g. means) or other basic estimates (e.g. regression coefficient) AND variation (e.g. standard deviation) or associated estimates of uncertainty (e.g. confidence intervals)
☐	☒	For null hypothesis testing, the test statistic (e.g. *F*, *t*, *r*) with confidence intervals, effect sizes, degrees of freedom and *P* value noted<br>*Give P values as exact values whenever suitable.*
☒	☐	For Bayesian analysis, information on the choice of priors and Markov chain Monte Carlo settings
☒	☐	For hierarchical and complex designs, identification of the appropriate level for tests and full reporting of outcomes
☐	☒	Estimates of effect sizes (e.g. Cohen's *d*, Pearson's *r*), indicating how they were calculated

Our web collection on statistics for biologists contains articles on many of the points above.

Software and code

Policy information about availability of computer code

Data collection	Blots pictures were acquired using the Chemi-Image System (Tanon 5200Multi).<br>All sequencing libraries were prepared in house and raw reads were generated on illumina high-throughtput sequencing platform with manufacture's instruction.
Data analysis	ImageJ (v1.6.0_24) was used for quantifying immunoblotting results.<br>FastP (v0.232), FeatureCounts (version 2.0.3), DESeq2 (v1.36.0), Bowtie2 (version 2.3.5.1), samtools (v1.9), MACS software (version 2.2.7.1), deepTools (v2.5.3), IGV (version 2.3.88), DiffBind (v3.5), homer (v4.11), clusterProfiler (4.10.0), Tbtools (v0.6), and R (v3.5) were used for RNA-seq and ChIP-seq data analysis. Detailed parameters of each of the programs are mentioned in relevant sections in Methods.

For manuscripts utilizing custom algorithms or software that are central to the research but not yet described in published literature, software must be made available to editors and reviewers. We strongly encourage code deposition in a community repository (e.g. GitHub). See the Nature Portfolio guidelines for submitting code & software for further information.

Data

Policy information about availability of data

All manuscripts must include a data availability statement. This statement should provide the following information, where applicable:

- Accession codes, unique identifiers, or web links for publicly available datasets
- A description of any restrictions on data availability
- For clinical datasets or third party data, please ensure that the statement adheres to our policy

The mass spectrometry data produced by this research were deposited to ProteomeXchage Consortium via the PRIDE partner repository with the dataset identifier PXD051126. The RNA-seq data, ChIP-seq data, and ATAC-seq data generated in this study were deposited to the NCBI GEO database under accession code GSE294824 (https://www.ncbi.nlm.nih.gov/geo/query/acc.cgi?acc=GSE294824), GSE294828 (https://www.ncbi.nlm.nih.gov/geo/query/acc.cgi?acc=GSE294828), and GSE294829 (https://www.ncbi.nlm.nih.gov/geo/query/acc.cgi?acc=GSE294829). Other previously published RNA-seq data used in this study are available in the NCBI SRA database under accession codes: SRX1500162 (https://www.ncbi.nlm.nih.gov/sra/?term=SRX1500162), SRX5197504 (https://www.ncbi.nlm.nih.gov/sra/?term=SRX5197504), SRX5636373 (https://www.ncbi.nlm.nih.gov/sra/?term=SRX5636373), SRX5636337 (SRX5636337), SRX1800518 (https://www.ncbi.nlm.nih.gov/sra/?term=SRX1800518), SRX5636385 (https://www.ncbi.nlm.nih.gov/sra/?term=SRX5636385). Previously published ATAC-seq and ChIP-seq data are under the accession codes: SRR10914733 (https://www.ncbi.nlm.nih.gov/sra/?term=SRR10914733), SRR10751615 (https://www.ncbi.nlm.nih.gov/sra/?term=SRR10751615), SRR10751619 (https://www.ncbi.nlm.nih.gov/sra/?term=SRR10751619), SRX1044777 (https://www.ncbi.nlm.nih.gov/sra/?term=SRX1044777), SRX189766 (https://www.ncbi.nlm.nih.gov/sra/?term=SRX189766), and SRX1620997 (https://www.ncbi.nlm.nih.gov/sra/?term=SRX1620997).

Research involving human participants, their data, or biological material

Policy information about studies with human participants or human data. See also policy information about sex, gender (identity/presentation), and sexual orientation and race, ethnicity and racism.

Reporting on sex and gender	N/A
Reporting on race, ethnicity, or other socially relevant groupings	N/A
Population characteristics	N/A
Recruitment	N/A
Ethics oversight	N/A

Note that full information on the approval of the study protocol must also be provided in the manuscript.

Field-specific reporting

Please select the one below that is the best fit for your research. If you are not sure, read the appropriate sections before making your selection.

[x] Life sciences [ ] Behavioural & social sciences [ ] Ecological, evolutionary & environmental sciences

For a reference copy of the document with all sections, see nature.com/documents/nr-reporting-summary-flat.pdf

Life sciences study design

All studies must disclose on these points even when the disclosure is negative.

Sample size	Three biological replicates were performed for RNA-seq and two biological replicates were performed for ChIP-seq in this study. For phenotype checking and enzyme activity assay, at lest two biological replicates were performed in this study.
Data exclusions	No data were excluded from analysis.
Replication	At lest two biological replicates were performed for experiments in this study as indicated in the figure legends.
Randomization	Samples were randomly allocated into experimental groups according to different mutants.
Blinding	Blinding was not relevant to our study.

Reporting for specific materials, systems and methods

We require information from authors about some types of materials, experimental systems and methods used in many studies. Here, indicate whether each material, system or method listed is relevant to your study. If you are not sure if a list item applies to your research, read the appropriate section before selecting a response.

Materials & experimental systems

n/a	Involved in the study	
☐	☒	Antibodies
☒	☐	Eukaryotic cell lines
☒	☐	Palaeontology and archaeology
☒	☐	Animals and other organisms
☒	☐	Clinical data
☒	☐	Dual use research of concern
☐	☒	Plants

Methods

n/a	Involved in the study	
☐	☒	ChIP-seq
☒	☐	Flow cytometry
☒	☐	MRI-based neuroimaging

Antibodies

Antibodies used

Antibodies used in this study:
anti-H3K9ac: Rabbit polyclonal antibody, Millipore (07-352). Dilution: 1:1000.
anti-H3: Rabbit polyclonal antibody, Abcam (ab1791). Dilution: 1:1000.
anti-Kbhb: Rabbit monoclonal antibody, PTM Bio (PTM-1201RM) Dilution: 1:1000.
anti-H3K9bhb: Rabbit monoclonal antibody, PTM Bio (PTM-1250RM) Dilution: 1:1000.
anti-H3K4ac: Rabbit monoclonal antibody, Abcam (ab176799), Dilution: 1:1000.
anti-Rabbit IgG: HRP goat anti-Rabbit IgG, Abbkine (A21020). Dilution: 1:10000.

Validation

anti-H3K9ac (Millipore, 07-352): https://www.merckmillipore.com/CN/zh/product/Anti-acetyl-Histone-H3-Lys9-Antibody,MM_NF-07-352
anti-H3 (Abcam, ab1791): https://www.abcam.com/histone-h3-antibody-nuclear-marker-and-chip-grade-ab1791.html
anti-Kbhb (PTM Bio, PTM-1201RM): https://www.ptmbio.com/products/anti-%CE%B2-hydroxybutyryllysine-rabbit-mab/PTM-1201RM.htm
anti-H3K9bhb(PTM Bio, PTM-1250RM): https://ptmbio.com/products/anti-%CE%B2-hydroxybutyryl-histone-h3-lys9-rabbit-mab/PTM-1250RM.htm
https://www.abcam.com/en-us/products/primary-antibodies/histone-h3-acetyl-k4-antibody-epr16596-chip-grade-ab176799
anti-Rabbit IgG: https://www.abbkine.com/product/hrp-goat-anti-rabbit-igg-a21020/

Dual use research of concern

Policy information about [dual use research of concern](https://www.nature.com/documents/nr-reporting-summary.pdf)

Hazards

Could the accidental, deliberate or reckless misuse of agents or technologies generated in the work, or the application of information presented in the manuscript, pose a threat to:

No	Yes	
☒	☐	Public health
☒	☐	National security
☒	☐	Crops and/or livestock
☒	☐	Ecosystems
☒	☐	Any other significant area

Experiments of concern

Does the work involve any of these experiments of concern:

No	Yes	
☒	☐	Demonstrate how to render a vaccine ineffective
☒	☐	Confer resistance to therapeutically useful antibiotics or antiviral agents
☒	☐	Enhance the virulence of a pathogen or render a nonpathogen virulent
☒	☐	Increase transmissibility of a pathogen
☒	☐	Alter the host range of a pathogen
☒	☐	Enable evasion of diagnostic/detection modalities
☒	☐	Enable the weaponization of a biological agent or toxin
☒	☐	Any other potentially harmful combination of experiments and agents

Plants

Seed stocks	The rice cultivars Wanxian98, Nipponbare, and ZH11 were individually used as the WT control in this study and are available in the state key laboratory for conservation and utilization of subtropical agro-bioresources, Guangxi University.
Novel plant genotypes	N/A
Authentication	N/A

ChIP-seq

Data deposition

☒ Confirm that both raw and final processed data have been deposited in a public database such as GEO.

☒ Confirm that you have deposited or provided access to graph files (e.g. BED files) for the called peaks.

Data access links<br>*May remain private before publication.*	All of raw data has been deposited to the NCBI GEO database under accession code GSE294828
Files in database submission	H3K9ac_WT_rep1<br>H3K9ac_WT_rep2<br>ChIP_CK_rep1<br>ChIP_CK_rep2<br>ChIP_IF_rep1<br>ChIP_IF_rep2<br>Infection_Input<br>hda705_rep1_K9bhb

	hda705_rep2_K9bhb<br>H3K9hb_ZH11_rep1<br>H3K9hb_ZH11_rep2<br>hda705_H3K9ac_rep1<br>hda705_H3K9ac_rep2<br>WT_H3K9ac_rep1<br>WT_H3K9ac_rep2<br>Input2<br>Kbhb_treat_input<br>ChIP_Kbhb_CK_rep1<br>ChIP_Kbhb_CK_rep2<br>ChIP_Kbhb_Treat_rep1<br>ChIP_Kbhb_Treat_rep2<br>srt2_rep1<br>srt2_rep2<br>WT_rep1<br>WT_rep2
Genome browser session (e.g. UCSC)	Not available.

Methodology

Replicates	Two biological replicates for each histone mark (H3K9bhb) in examined tissues.
Sequencing depth	About 20 million pair-end (2x150bp) raw reads on average for each experiment.
Antibodies	anti-H3K9bhb (PTM Bio, PTM-1250RM) ; anti-H3K9ac (Millipore, 07-352)
Peak calling parameters	macs2 callpeak -f BAMPE -B -q 0.05 -g 3.6e+8
Data quality	Alll identified peaks in the study were called with a qval threshold of 0.05(FDR 5%).
Software	FastP (v0.232), Bowtie2 (version 2.3.5.1), samtools (v1.9), MACS software (version 2.2.7.1), deepTools (v2.5.3), IGV (version 2.3.88), DiffBind (v3.5), homer (v4.11).
